# MARCH2 suppresses odontoblast differentiation by polyubiquitinating PTPRD

**DOI:** 10.1038/s41368-025-00407-2

**Published:** 2026-01-10

**Authors:** Hao Feng, Jiaxin Niu, Zhi Chen, Guobin Yang, Guohua Yuan

**Affiliations:** 1 State Key Laboratory of Oral & Maxillofacial Reconstruction and Regeneration, Key Laboratory of Oral Biomedicine Ministry of Education, Hubei Key Laboratory of Stomatology, School & Hospital of Stomatology, Wuhan University, Wuhan, China; 2 Frontier Science Center for Immunology and Metabolism, Wuhan University, Wuhan, China; 3 Hubei Provincial Key Laboratory of Developmentally Originated Disease, Wuhan University, Wuhan, China

**Keywords:** Differentiation, Ubiquitin ligases

## Abstract

Dentin, the main component of dental hard tissues, is produced by differentiated odontoblasts. How odontoblast differentiation is regulated remains understudied. Here, we screen that the expression of *membrane-associated RING finger protein 2* (*March2*) is the highest among all March family members, with an increasing trend during odontoblast differentiation. In mouse incisors and molars, MARCH2 is moderately expressed in the undifferentiated dental papilla cells and strongly expressed in the odontoblasts. Knockdown and overexpression experiments demonstrate that MARCH2 inhibits odontoblastic differentiation of mouse dental papilla cells (mDPCs). Additionally, both *March2* deficient mice and mice with odontoblast specific knockdown of *March2* exhibit the phenotype of increased dentin thickness, accelerated dentin deposition as well as elevated expression levels of odontoblast markers compared with control littermates. Therefore, MARCH2 plays an inhibitory role in odontoblast differentiation. Mechanistically, MARCH2 interacts with protein tyrosine phosphatase receptor delta (PTPRD) and facilitates its K27-linked polyubiquitination and subsequent degradation, which is dependent on the ligase activity of MARCH2. The presence of MARCH2 promotes the translocation of PTPRD from the cell membrane to the lysosome, thereby enhancing its degradation via the lysosomal pathway. Further experiments show that knockdown of endogenous *Ptprd* impairs odontoblastic differentiation of mDPCs. *Ptprd* and *March2* double knockdown in mDPCs apparently reversed the enhanced odontoblastic differentiation by knockdown of *March2* alone, indicating that MARCH2 inhibits odontoblastic differentiation by promoting PTPRD degradation. This study unveils a novel mechanism where an E3 ubiquitin ligase regulates odontoblast differentiation through post-translational modification of a membrane protein, highlighting a promising direction for future exploration.

## Introduction

As the main constituent of dental hard tissues, dentin supports the outer enamel and protects the inner dental pulp. At the bell stage of tooth development, the mesenchymal cells adjacent to the dental epithelium differentiate into odontoblasts and initiate dentin production. Abnormalities of odontoblast differentiation can lead to defects in dentin formation.^1^

Membrane proteins are essential components of the cell membrane and play pivotal roles in various physiological processes.^2^ Notably, some membrane proteins participate in the differentiation processes of diverse tissue or cell types,^3,4^ including odontoblast differentiation.^5^ Ubiquitination, a form of post-translational modification, modulates the conformation, stability, and localization of membrane proteins, thereby influencing their function.^6,7^ The membrane-associated RING-CH (MARCH) E3 ubiquitin ligases are a family of RING-type E3 ligases, comprising 11 members, named membrane-associated RING finger protein 1 (MARCH1) to MARCH11.^8^ All members of the family except MARCH7 and MARCH10 contain at least one transmembrane domain and are located at the cell membranes or organelle membranes,^9^ and function through controlling the stability and activity of various cellular membrane proteins.^10^ Members of the MARCH family play critical roles in many developmental processes. For instance, MARCH1 mediates the removal of major histocompatibility complex class II (MHCII) and cluster of differentiation 86 (CD86) on dendritic cells residing in the lymph nodes, thereby promoting the differentiation of Type 2T helper cells.^11^ MARCH2 targets *Xenopus* dishevelled (DSH) for ubiquitin-mediated degradation, counteracting Wnt signaling and thus controlling proper head development.^8^ MARCH10 is involved in spermiogenesis by regulating the assembly and maintenance of the flagella in developing spermatids.^12^ However, the role of MARCH family members in odontoblast differentiation and dentinogenesis as well as the membrane proteins targeted by them remain unclear.

In this study, we show that MARCH2 exhibits the highest expression level among all MARCH family members during odontoblastic differentiation of mouse dental papilla cells (mDPCs). Knockdown and overexpression experiments reveal that MARCH2 inhibits odontoblastic differentiation of mDPCs. Using *March2* deficient mice and mice with odontoblast specific knockdown of *March2*, we demonstrate that in mouse molars and incisors, MARCH2 exerts an inhibitory effect on dentinogenesis and odontoblast differentiation. Mechanistically, MARCH2 targets the membrane protein, protein tyrosine phosphatase receptor delta (PTPRD) for polyubiquitination, which promotes subsequent lysosomal degradation of PTPRD.

## Results

### MARCH2 is expressed during odontoblast differentiation

To screen the expression levels of MARCH family members during odontoblastic differentiation, real-time quantitative polymerase chain reaction (RT-qPCR) was conducted using mDPCs cultured in differentiation medium for 0 day (d), 3 d and 5 d. The results revealed that, among all MARCH members, the expression of *March2* was the highest with an increasing trend during odontoblastic differentiation (Supplementary Fig. S1a, b). Next, Western blot (WB) analysis was performed. Similarly, the results showed a gradual increase of MARCH2 protein during this process (Supplementary Fig. S1c, d). Meanwhile, immunocytochemistry (ICC) also revealed that the expression of MARCH2 increased with odontoblastic differentiation, and MARCH2 was present in the subcellular compartments of the cytoplasm and cell membrane but not in the nucleus (Supplementary Fig. S1e, f). Analysis of single-cell sequencing data from tooth samples at postnatal day 3.5 (PN3.5)^13^ showed that *March2* exhibited the highest average mRNA level within the odontoblast subpopulation among all dental populations (Supplementary Fig. S2a–d).

**Fig. 1:**
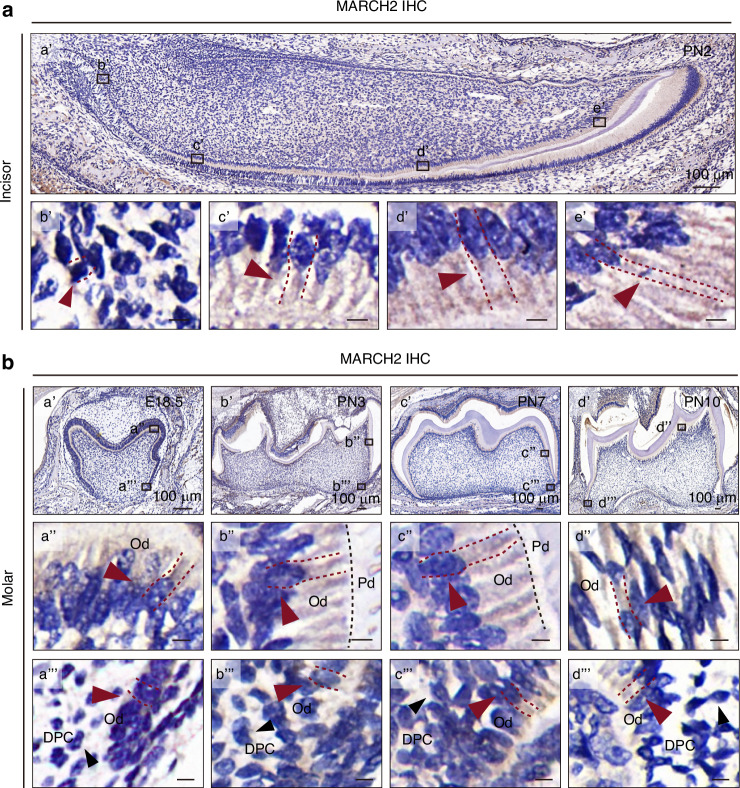
The expression pattern of MARCH2 during odontoblast differentiation in vivo. **a** Immunohistochemistry (IHC) of MARCH2 in postnatal day 2 (PN2) mouse incisor. b’, c’, d’, and e’ are enlarged views of the rectangles in a’. Red arrows and red dashed lines indicate dental papilla cells or odontoblasts. **b** IHC of MARCH2 in mouse molars from embryonic day 18.5 (E18.5) to PN10. a”-d” and a”’-d”’ are enlarged views of the rectangles in a’-d’. Red arrows and red dashed lines mark the odontoblasts. Black arrows mark the dental papilla cells. The black dotted lines represent the boundaries between pre-dentin and odontoblast layer. Od odontoblasts, DPC dental papilla cell, Pd pre-dentin. Except for the labeled scale bars, all others are 5 µm

To further explore the in vivo expression of MARCH2, immunohistochemistry (IHC) was performed on PN2 mouse incisors which encompass all stages of odontoblast differentiation within a single section (Fig. 1a a’). The results showed that MARCH2 exhibited a moderate level in the undifferentiated dental papilla cells (DPCs) and polarizing odontoblasts (Fig. 1a b’, c’), and an intense level in the secretory and mature odontoblasts (Fig. 1a d’, e’). It was noted that MARCH2 was extensively distributed except the nucleus compartment, consistent with its reported subcellular localization at endosomes, lysosomes and the cell membrane.^14^ Meanwhile, IHC of MARCH2 was also performed in mouse molars. The results showed that MARCH2 was moderately expressed in the undifferentiated DPCs and strongly expressed in the odontoblasts at embryonic day 18.5 (E18.5), PN3, PN7, and PN10 (Fig. 1b a’–d’).

In addition, ameloblasts were also positive for MARCH2 (Fig. 1a, b), but no positive signals were observed in the negative controls (Supplementary Fig. S3).

### MARCH2 suppresses odontoblastic differentiation of mDPCs in vitro

**Fig. 2:**
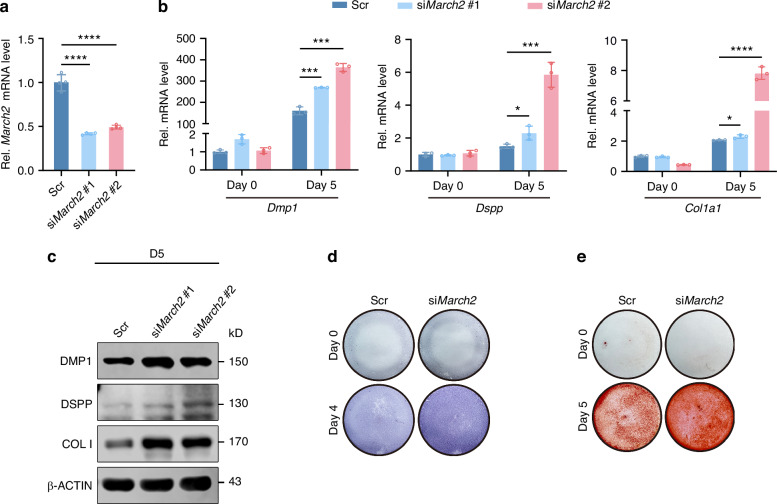
Knockdown of *March2* promotes the odontoblastic differentiation of mouse dental papilla cells (mDPCs) in vitro. **a** The knockdown efficiency of two *March2* small interfering RNAs (siRNAs) in mDPCs was assessed by real-time quantitative PCR (RT-qPCR) (*n* = 4). **b** The effects of *March2* knockdown on mRNA levels of *Dmp1*, *Dspp* and *Col1a1* after differentiation induction of mDPCs for 5 days (5 d) (*n* = 3). **c** The effects of *March2* knockdown on protein levels of DMP1, DSPP and COL I after differentiation induction of mDPCs for 5 d. **d** The effects of *March2* knockdown on the alkaline phosphatase (ALP) activity detected by ALP staining. **e** The effects of *March2* knockdown on the formation of mineralized nodule assessed by Alizarin Red S (ARS) staining. Scr Scramble siRNA, D differentiation induction. The statistical difference was analyzed by one-way ANOVA with Tukey’s post hoc test (**a**, **b**), where ^*^P < 0.05; ^***^P < 0.001; ^****^P < 0.000 1

To investigate the role of MARCH2 in odontoblastic differentiation of mDPCs, knockdown experiments were performed using small interfering RNAs (siRNAs), which result in a significant reduction of *March2* mRNA as assessed by RT-qPCR (Fig. 2a). The levels of odontoblast marker genes including *dentin matrix protein 1* (*Dmp1*), *dentin sialophosphoprotein* (*Dspp*) and *collagen type I alpha 1 chain* (*Col1a1*) were significantly increased in the *March2* knockdown cells after culture in differentiation medium as shown by RT-qPCR (Fig. 2b). In accordance, WB analysis verified that the protein levels of DMP1, DSPP and collagen type I (COL I) were elevated in the *March2* knockdown group (Fig. 2c; Supplementary Fig. S4a). Alkaline phosphatase (ALP) staining and alizarin red S (ARS) staining showed that both ALP activity (Fig. 2d) and mineralized nodule formation (Fig. 2e; Supplementary Fig. S4b) were increased upon *March2* knockdown.

Next, to further confirm its role, MARCH2 was overexpressed in mDPCs. The results showed that, after an increase of MARCH2 protein by 13.5-fold (Supplementary Fig. S5a), mRNA and protein levels of DMP1, DSPP and COL I (Supplementary Fig. S5b–d) as well as ALP activity (Supplementary Fig. S5e) and calcified nodule formation (Supplementary Fig. S5f, g) were all downregulated compared with the control group.

Therefore, both the knockdown and overexpression experiments support that MARCH2 exerts an inhibitory role in the odontoblastic differentiation of mDPCs.

### *March2* deletion leads to increased dentin formation in vivo

**Fig. 3:**
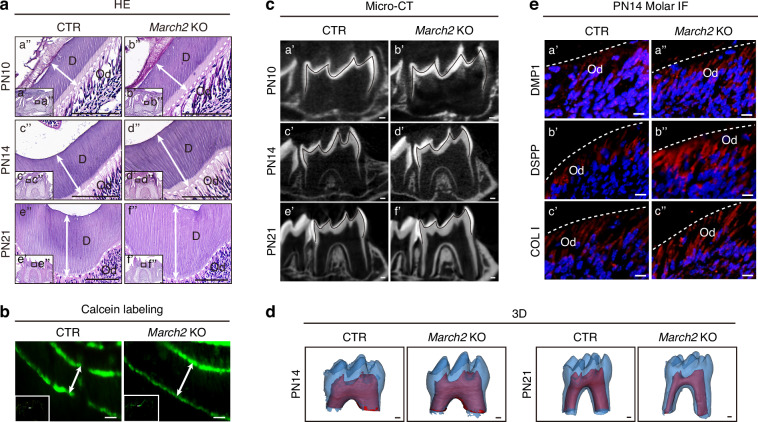
*March2* deficient mice exhibit enhanced odontoblast differentiation and dentinogenesis. **a** Hematoxylin and Eosin (H&E) staining of molars from control littermates and *March2* deficient mice at PN10 (a’, b’), PN14 (c’, d’) and PN21 (e’, f’). The white dotted lines represent the boundaries between dentin and odontoblast layer. The white arrows represent the widths of dentin. a”-f” are magnified views of the black box in a’-f’. Scale bar, 100 μm. **b** Calcein labeling was performed to compare the dentin deposition rate between control littermates and *March2* deficient mice. The white arrows indicate the dentin deposited in the period between drug administrations. Scale bar, 10 μm. **c** Micro-computed tomography (micro-CT) images of the mandibular molars from control littermates and *March2* deficient mice at PN10 (a’, b’), PN14 (c’, d’) and PN21 (e’, f’). The black lines delineate the interface between the dentin and enamel. Scale bar, 100 μm. **d** Three-dimensional (3D) reconstruction of the first mandibular molars from control littermates and *March2* deficient mice at PN14 and PN21. Scale bar, 100 μm. **e** Immunofluorescent (IF) staining of the odontoblast markers DMP1 (a’, a”), DSPP (b’, b”) and COL I (c’, c”) in molars of control littermates (a’, b’, c’) and *March2* deficient (a”, b”, c”) mice at PN14. Scale bar, 10 μm. CTR control littermates, *March2* KO *March2* deficient, D dentin, Od odontoblasts

To observe the in vivo function of MARCH2 in dentin formation, *March2* deficient mice were generated using CRISPR/Cas9 technology (Supplementary Fig. S6a). Sanger sequencing of the *March2* deficient mice revealed a 7 bp deletion in exon 3, leading to a frameshift and premature termination of translation of MARCH2 protein (Supplementary Fig. S6b). The successful inactivation of *March2* was further confirmed by IHC analysis (Supplementary Fig. S6c). Hematoxylin and eosin (H&E) staining revealed that the widths of dentin in *March2* deficient mice were significantly increased compared with those in control littermates at PN10, PN14 and PN21 (Fig. 3a; Supplementary Fig. S7a). Additionally, double-calcein labeling experiments suggested an accelerated rate of dentin deposition in *March2* deficient mice (Fig. 3b; Supplementary Fig. S7b). Micro-computed tomography (micro-CT) confirmed increased dentin thickness in the molars of *March2* deficient mice (Fig. 3c). Three-dimensional (3D) reconstruction analysis of PN14 and PN21 molars revealed increased dentin volume in *March2* deficient mice, while the enamel volume remained comparable to that of control littermates (Fig. 3d; Supplementary Fig. S7c). Micro-CT analysis of incisors also revealed increased dentin thickness in *March2* deficient mice compared with control littermates (Supplementary Fig. S8a). Furthermore, double-calcein labeling experiments indicated accelerated dentin deposition rate in the incisors of *March2* deficient mice (Supplementary Fig. S8b, c). These findings indicate that MARCH2 inhibits dentinogenesis in vivo.

To verify the in vivo effects of MARCH2 on the differentiation of odontoblasts, the cell type responsible for dentin formation, immunofluorescence (IF) was performed to assess the expression levels of the odontoblast markers including DMP1, DSPP and COL I. The results demonstrated that all these markers were upregulated in the odontoblast layer of *March2* deficient mice at PN14 (Fig. 3e; Supplementary Fig. S7d). Therefore, deletion of *March2* leads to enhanced dentinogenesis and odontoblast differentiation in mouse molars.

### Mice with specific knockdown of *March2* in the odontoblast layer recapitulates the dentin phenotype of *March2* deficient mice

**Fig. 4:**
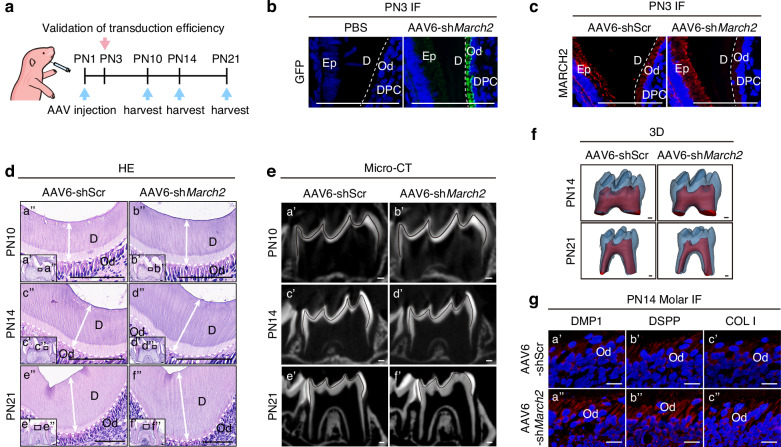
In vivo knockdown of *March2* in odontoblasts leads to increased odontoblast differentiation and dentin formation in mice. **a** Schematic diagram showing time points for AAV injection and tissue collection. Mice were injected with AAV6-shScr or AAV6-sh*March2* at PN1. Samples were collected at PN3 to assess the transduction efficiency, and harvested at PN10, PN14 and PN21 to evaluate the phenotype. **b** Mandibles from PN3 mice after injection with AAV6-sh*March2* or PBS were processed for frozen sectioning, and GFP fluorescence was examined. **c** IF staining of MARCH2 in molars of PN3 mice in AAV6-shScr or AAV6-sh*March2* group. **d** HE staining of molars in the AAV6-shScr and AAV6-sh*March2* group at PN10 (a’, b’), PN14 (c’, d’) and PN21 (e’, f’). a”-f” are magnified views of the black boxes in a’-f’. The white dotted lines in **b**, **c**, **d** represent the boundaries between dentin and the odontoblast layer. The white arrows in **d** represent the widths of dentin. **e** Micro-CT images of the mandibular molars at PN10 (a’, b’), PN14 (c’, d’) and PN21 (e’, f’) after AAV6-shScr or AAV6-sh*March2* injection. The black lines delineate the interface between the dentin and enamel. **f** 3D reconstruction of the first mandibular molars from mice at PN14 and PN21 after following injection with AAV6-shScr or AAV6-sh*March2*. **g** IF staining of the odontoblast markers DMP1 (a’, a”), DSPP (b’, b”) and COL I (c’, c”) in molars of PN14 mice of AAV6-shScr (a’, b’, c’) or AAV6-sh*March2* (a”, b”, c”) group. Ep epithelium, Od odontoblasts, DPC dental papilla cells, D dentin. Scale bar, 100 μm

Systemic *March2* inactivation may introduce confounding effects from other tissues. To achieve specific knockdown of *March2* in the odontoblast layer, we constructed an adeno-associated virus serotype 6 (AAV6) vector expressing a short hairpin RNA (shRNA) targeting *March2* under the control of the mouse *Dmp1* promoter (designated as AAV6-sh*March2*), which was locally injected into the mandibular molar germs (Fig. 4a) as previously reported.^1^ This vector also includes a GFP reporter to enable tracking of transduction efficiency. 2 days after injection, GFP was detected in the odontoblast layer but not other cell types of AAV6-sh*March2*-injected molars (Fig. 4b) and the percentage of GFP-positive odontoblasts reached 83% (Supplementary Fig. S9a). IF analysis further showed reduced MARCH2 expression within the odontoblast layer, whereas its expression in the dental epithelium and other cells remained unchanged (Fig. 4c), indicating successful specific knockdown of *March2* in the odontoblast layer. H&E staining confirmed that the widths of dentin in AAV6-sh*March2* group were increased compared with those in AAV6-Scramble shRNA (AAV6-shScr) group at PN10, PN14 and PN21 (Fig. 4d; Supplementary Fig. S9b). Micro-CT analysis revealed that AAV6-sh*March2*-injected molars exhibited increased dentin thickness (Fig. 4e). 3D reconstruction of PN14 and PN21 molars demonstrated increase of dentin volume in these mice, while enamel volume remained similar to that of AAV6-shScr-injected control molars (Fig. 4f; Supplementary Fig. S9c). Moreover, IF showed upregulated expression of DMP1, DSPP and COL I in the odontoblast layer of AAV6-sh*March2* group at PN14 (Fig. 4g; Supplementary Fig. S9d). Together, these results indicate that specific knockdown of *March2* in the odontoblast layer enhances odontoblast differentiation and dentinogenesis.

### MARCH2 polyubiquitinates PTPRD and promotes its lysosomal degradation

To explore the underlying mechanisms by which MARCH2 influences odontoblast differentiation, the bioinformatic platforms including UbiBrowser (http://ubibrowser.bio-it.cn/) and BioGrid (https://www.biogrid.org.au/) were used to analyze potential targets of MARCH2. After screening, PTPRD, a member of the Leukocyte Common Antigen-Related Receptor Protein Tyrosine Phosphatase (LAR-RPTP) family, attracted our attention as a potential candidate target of MARCH2. As a membrane protein, PTPRD has been found to be involved in diverse developmental processes, including neurogenesis,^15^ gliogenesis,^3^ and adipogenesis.^16^

**Fig. 5:**
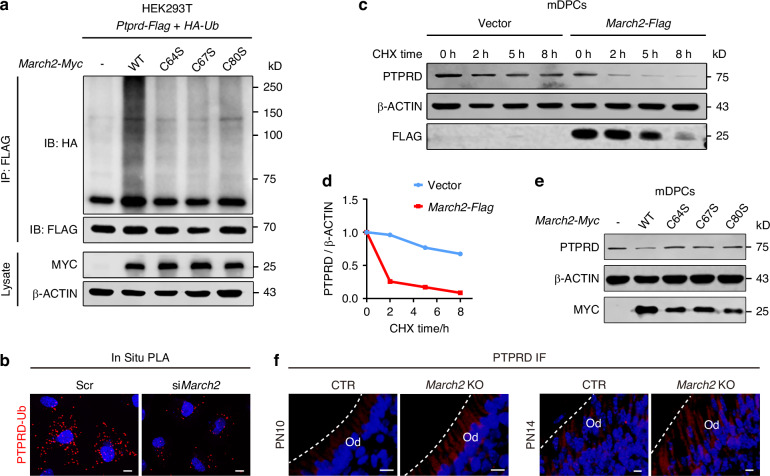
MARCH2 promotes the polyubiquitination of PTPRD and its subsequent degradation. **a** Wild-type *March2-Myc* or its ligase-inactive mutants (*C64S*, *C67S*, and *C80S*) were overexpressed together with *Ptprd-Flag* and *HA-ubiquitin* in human embryonic kidney 293T (HEK293T) cells. Co-immunoprecipitation (Co-IP) assays show that MARCH2 ubiquitinated PTPRD dependent of its ligase activity. **b** The level of ubiquitinated PTPRD is reduced following *March2* knockdown in mDPCs, as demonstrated by in situ proximity ligation assay (PLA) with anti-PTPRD and anti-ubiquitin antibodies. Red punctate fluorescence indicates the physical proximity of two proteins. **c** Cycloheximide (CHX) chase assays demonstrate that overexpressed MARCH2 decreases the half-life of endogenous PTPRD protein in cultured mDPCs. Cells were treated with CHX for 0, 2, 5 and 8 h (h) before harvest for WB analysis. **d** Quantitative analysis of the relative PTPRD levels at different time points in (**c**), suggesting that MARCH2 promotes the degradation of PTPRD. **e** Wild-type *March2-Myc* or its ligase-inactive mutants (*C64S*, *C67S*, and *C80S*) were overexpressed in mDPCs. WB analysis shows that wild-type MARCH2 reduced the endogenous protein level of PTPRD but its ligase-inactive mutants did not show this effect. **f** IF staining shows a higher level of PTPRD protein in the molars of *March2* deficient mice at PN10 and PN14. The white dashed line represents the junction between odontoblasts and dentin. Scr Scramble siRNA, CTR control littermates, *March2* KO *March2* deficient, Od odontoblasts. Scale bar, 10 μm

To confirm whether PTPRD acted as the substrate of MARCH2 during odontoblast differentiation, several lines of experiments were conducted. Firstly, the physical interaction between MARCH2 and PTPRD, which is the prerequisite for PTPRD as the substrate of MARCH2, was analyzed in cultured cell types using co-immunoprecipitation (co-IP) assays. The results showed that overexpressed MARCH2 and PTPRD were able to pull down each other in human embryonic kidney 293 T (HEK293T) cells (Supplementary Fig. S10a, b). Furthermore, MARCH2 was found to interact with endogenous PTPRD in mDPCs (Supplementary Fig. S10c). Meanwhile, in situ proximity ligation assay (PLA) demonstrated the physical proximity between the overexpressed MARCH2 and endogenous PTPRD in mDPCs (Supplementary Fig. S10d). Ubiquitination modification includes polyubiquitination and monoubiquitination according to the ubiquitin chains.^17^ Co-IP assays showed that wild-type MARCH2 increased the polyubiquitination of PTPRD but its ligase-inactive mutants (*March2* C64S, C67S, and C80S) did not (Fig. 5a). Meanwhile, knockdown of *March2* in mDPCs reduced the ubiquitination of endogenous PTPRD as assessed by in situ PLA (Fig. 5b). Therefore, MARCH2 polyunbiquitinates PTPRD.

Polyubiquitination leads to different outcomes for the substrates, with protein degradation being the most frequent.^18^ Therefore, we examined whether MARCH2 targets PTPRD for degradation. Our results showed that MARCH2 overexpression did not affect the mRNA level of *Ptprd*, ruling out the possibility of transcriptional regulation of PTPRD by MARCH2. (Supplementary Fig. S11). Cycloheximide (CHX, a protein synthesis inhibitor) chase assays showed that MARCH2 overexpression indeed increased the degradation velocity of PTPRD in mDPCs (Fig. 5c, d). Additionally, wild-type MARCH2, but not its ligase-inactive mutants, promoted the degradation of endogenous PTPRD (Fig. 5e; Supplementary Fig. S12a), suggesting that the increased degradation of PTPRD caused by MARCH2 is dependent on the ligase activity of MARCH2. Consistently, IF showed that PTPRD protein level was elevated in the odontoblasts of *March2* deficient mice at PN10 and PN14 (Fig. 5f; Supplementary Fig. S12b). To define ubiquitination type of PTPRD mediated by MARCH2, we co-transfected PTPRD with ubiquitin mutants that contain only a single lysine residue. This analysis revealed that MARCH2 specifically promoted K27-linked polyubiquitination of PTPRD. By cotransfection of PTPRD with ubiquitin mutants where a single lysine residue was substituted with arginine, we found that MARCH2 could not promote the polyubiquitination of PTPRD only when K27 was mutated to arginine (Supplementary Fig. S13). Therefore, MARCH2 facilitates the K27-linked polyubiquitination of PTPRD and its subsequent degradation.

**Fig. 6:**
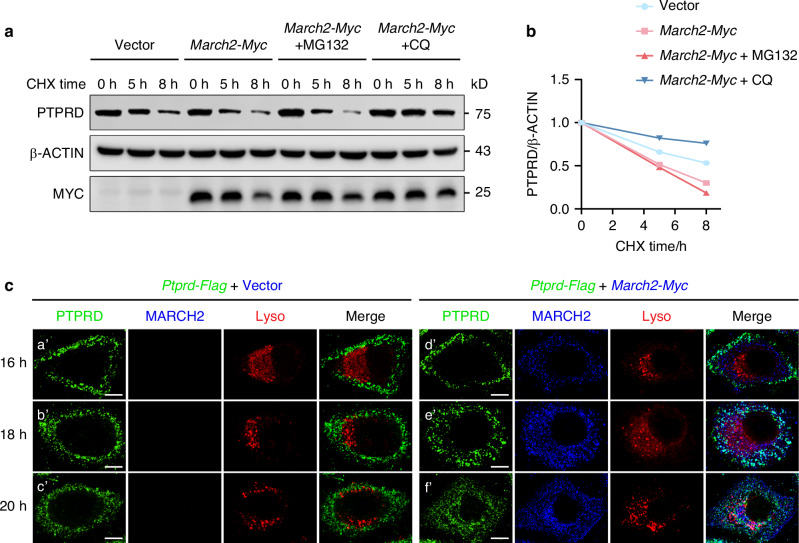
MARCH2 promotes the lysosomal degradation of PTPRD. **a** Chloroquine (CQ), a lysosome inhibitor, suppresses the degradation of endogenous PTPRD by overexpressed MARCH2 in mDPCs, while MG132, a proteasome inhibitor, does not. Cells were cultured with CHX and the inhibitors for up to 8 h before harvest as indicated, with MG132 and CQ administered at working concentrations of 40 nM and 10 mol/L, respectively. **b** Quantitative analysis of the relative PTPRD levels at different time points in (**a**). **c** MARCH2 facilitates the translocation of PTPRD from the cell membrane to intracellular area where PTPRD shows co-localization with lysosomes. Henrietta Lacks (Hela) cells were transfected with *Ptprd-Flag* plasmid alone or together with *March2-Myc* plasmid, and cultured for 16 h, 18 h or 20 h. The cells were incubated with LysoTracker Red (200 mol/L) for 1 h prior to collection. After collection, cells were fixed with 4% paraformaldehyde, and then stained with mouse anti-FLAG and rabbit anti-MYC antibodies. PTPRD and MARCH2 were visualized using Alexa Fluor 488- and Alexa Fluor 647-conjugated secondary antibodies, respectively. Scale bar, 10 μm

To investigate the degradation pathway of PTPRD, we used inhibitors of proteasomal and lysosomal pathways and cultured for different time intervals. The results showed that application of Chloroquine (CQ, a lysosome inhibitor) but not MG132 (a proteasome inhibitor) attenuated MARCH2-induced PTPRD degradation, suggesting that MARCH2 triggered lysosomal rather than proteasomal degradation of PTPRD (Fig. 6a, b). When PTPRD alone was overexpressed, it was predominantly present on the cell membrane (Fig. 6c a’–c’). However, when MARCH2 was concurrently overexpressed, PTPRD was progressively translocated to the cytoplasm and exhibited colocalization with lysosome (Fig. 6c d’–f’), indicating that MARCH2 promotes the translocation of PTPRD from the cell membrane to the intracellular lysosomes. These findings demonstrated that MARCH2 facilitates K27-linked polyubiquitination of PTPRD, thereby directing its degradation through the lysosomal pathway.

### PTPRD is expressed in the odontoblast layer and promotes the odontoblastic differentiation of mDPCs in vitro

To unveil the role of PTPRD during odontoblast differentiation, the expression of PTPRD in mouse teeth was first investigated. IHC showed that PTPRD was expressed in the odontoblast layer of both incisors and molars in mice (Supplementary Fig. S14). *Ptprd* was then knocked down in mDPCs using two siRNAs which gave rise to over 70% reduction of *Ptprd* mRNA as shown by RT-qPCR (Supplementary Fig. S15a). Compared with the control group transfected with scramble siRNA, the mRNA levels of *Dmp1*, *Dspp* and *Col1a1* were significantly reduced in the *Ptprd* knockdown group after culture in differentiation medium (Supplementary Fig. S15b). Consistently, WB analysis demonstrated reduced protein levels of DMP1, DSPP and COL I (Supplementary Fig. S15c, d). Additionally, ALP and ARS staining revealed decreased ALP activity (Supplementary Fig. S15e) and reduced formation of mineralized nodules (Supplementary Fig. S15f, g) following *Ptprd* knockdown. These results indicate that PTPRD plays a positive role in the odontoblastic differentiation of mDPCs.

### MARCH2 inhibits odontoblastic differentiation via PTPRD

**Fig. 7:**
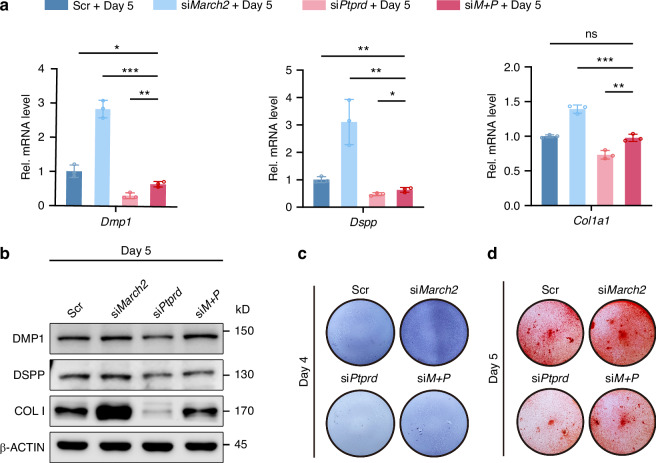
*Ptprd* knockdown reverses the enhanced odontoblastic differentiation of mDPCs by *March2* deficiency. **a** RT-qPCR shows the mRNA levels of *Dmp1*, *Dspp* and *Col1a1* in mDPCs cultured in the differentiation medium for 5 d following transfection with siRNAs targeting *March2* and *Ptprd* each or both (*n* = 3). **b** WB analysis shows the protein levels of DMP1, DSPP and COL I in mDPCs cultured for 5 d in the differentiation medium following transfection with *March2* siRNA or *Ptprd* siRNA or both. **c** The increase in ALP activity in differentiation-induced cells due to *March2* knockdown is partially reversed by the simultaneous knockdown of *Ptprd* and *March2*. **d** ARS staining indicates that *March2* knockdown promotes mineralized nodule formation in differentiation-induced cells, an effect that is abolished by the simultaneous knockdown of *Ptprd* and *March2*. D, differentiation induction. The statistical difference was analyzed by one-way ANOVA with Dunnett’s post hoc test (**a**), where ns denotes not significant; ^*^P < 0.05; ^**^P < 0.01; ^***^P < 0.001

To determine whether PTPRD was the key mediator of the role of MARCH2 during odontoblast differentiation, simultaneous knockdown of *March2* and *Ptprd* in mDPCs was performed. *March2* knockdown led to upregulation of both mRNA and protein levels of DMP1, DSPP and COL I, which were remarkably reversed by concurrent knockdown of *Ptprd* shown by RT-qPCR and WB analysis (Fig. 7a, b; Supplementary Fig. S16a). Additionally, concurrent knockdown of *Ptprd* partially reversed the alterations in ALP activity (Fig. 7c) and mineralized nodules formation (Fig. 7d; Supplementary Fig. S16b) induced by *March2* knockdown. These findings suggest that MARCH2 inhibits odontoblastic differentiation through PTPRD.

## Discussion

E3 ligases target a broad spectrum of substrates to regulate fundamental developmental processes, including cell differentiation.^19^ In this study, we identified MARCH2, a membrane-associated E3 ligase, plays a negative regulatory role in odontoblast differentiation and dentinogenesis through mediating the polyubiquitination and lysosomal degradation of the membrane protein PTPRD. Several other E3 ubiquitin ligases including smad ubiquitination regulatory factor 1 (SMURF1), murine double minute 2 (MDM2), WW domain-containing E3 ubiquitin protein ligase 2 (WWP2) and carboxyl-terminus of Hsc70 interacting protein (CHIP) have been reported to play pivotal roles in modulating odontoblast differentiation and dentinogenesis.^20–25^ However, the substrates of these E3 ligases are all cytoplasmic or nuclear proteins, different from the present study with a membrane protein as the substrate of the E3 ubiquitin ligase MARCH2.

Among all E3 ubiquitin ligases localized to cellular and organelle membranes, MARCH family members comprise a substantial proportion.^8^ Based on our preliminary experiments, MARCH2 was screened out since its expression was the highest among all MARCH family members and increased during in vitro odontoblastic differentiation of mDPCs. IHC analysis of incisors at PN2 and molars at E18.5, PN3, PN7 and PN10 revealed that the expression of MARCH2 was moderate in the undifferentiated DPCs and strong in the odontoblasts in vivo. The expression pattern of MARCH2 suggests a possible role of it during odontoblast differentiation.

MARCH2 has been reported to be involved in various physiological processes including vesicular trafficking^26,27^ and autophagy,^28,29^ as well as in several pathological processes, such as myocardial ischemia^30^ and allergic airway inflammation.^9^ It is also involved in the developmental process of the *Xenopus* head.^8^ To investigate the role of MARCH2 during the odontoblastic differentiation of mDPCs, a series of knockdown and overexpression experiments were performed in mDPCs. The results of these in vitro experiments collectively demonstrated an inhibitory role of MARCH2 during this process. Furthermore, *March2* deficient mice and mice with odontoblast specific knockdown of *March2* exhibited increased dentin thickness, accelerated dentin deposition rate as well as elevated expression levels of odontoblast markers in both incisors and molars compared with control mice, consistent with the findings of in vitro experiments. The high expression of MARCH2 in odontoblasts appears paradoxical given its inhibitory role in odontoblast differentiation. However, we speculate that MARCH2 act as a negative regulatory mechanism to restrain pro-differentiation signals, thereby preventing excessive odontoblast differentiation and maintaining balanced dentin formation. A similar mechanism is seen in osteoblasts, where the E3 ligase SMURF1 targets runt-related transcription factor 2 (RUNX2) for degradation, thereby delaying osteoblast differentiation and limiting excessive bone formation.^31^

Based on phylogenetic analysis, the MARCH family is classified into four subgroups, with MARCH2 and MARCH3 sharing significant similarity and being placed in the same subgroup. Previous studies have demonstrated that MARCH2 and MARCH3 exhibit redundant roles in regulating allergic airway inflammation.^9^ However, during odontoblastic differentiation, *March3* is hardly expressed in the dental papilla (Supplementary Figs. S1a, b, S2e), thereby ruling out a compensatory role of it for MARCH2 in dentinogenesis.

As a member of the MARCH family, MARCH2 typically targets substrates localized at the cell membrane and organelle membranes, promoting their ubiquitination and subsequent degradation.^9,27,28,30^ This study revealed that MARCH2 interacted with PTPRD, a membrane protein, and promoted its polyubiquitination and degradation dependent of the ubiquitin ligase activity of MARCH2. *March2* deficient mice exhibited elevated PTPRD protein level, supporting that the degradation of PTPRD is mediated by MARCH2. Further experiments confirmed that MARCH2 promoted the K27-linked polyubiquitylation of PTPRD. Therefore, MARCH2 targets PTPRD for the K27-linked polyubiquitination and subsequent degradation. Likewise, MARCH2 has been reported to interact with interleukin-5 receptor alpha (IL-5Rα), mediating its K27-linked polyubiquitination and subsequent degradation.^9^ Notably, the residual ubiquitination of PTPRD observed in *March2-Myc*-untransfected groups may be attributed to functional redundancy between MARCH2 and other E3 ubiquitin ligases. Ubiquitination mediated protein degradation has been found to occur through proteasomal and/or lysosomal pathways.^32^ Application of CQ, rather than MG132, rescued MARCH2-induced degradation of endogenous PTPRD, indicating that MARCH2 promotes PTPRD degradation through the lysosomal pathway. Additionally, we found that overexpressed MARCH2 facilitated translocation of PTPRD from cell membrane to intracellular area where PTPRD showed colocalization with lysosome, supporting the above finding of MARCH2-mediated lysosomal degradation of PTPRD.

Multiple receptor proteins have been found to be involved in the process of odontoblast differentiation.^33–35^ PTPRD, as a member of the LAR-RPTP family, is localized on the cell membrane and influences signal transduction by mediating tryosine residues dephosphorylation of intracellular proteins.^36^ In this study, knockdown of *Ptprd* impaired the mRNA and protein levels of DMP1, DSPP and COL I, as well as ALP activity and mineralized nodule formation ability, revealing that PTPRD was able to promote the odontoblastic differentiation of mDPCs. Previous studies have shown that PTPRD facilitates the differentiation of mesenchymal progenitor cell into adipocytes by modulating FYN tyrosine phosphorylation levels.^16^ Furthermore, PTPRD has been reported to specifically dephosphorylate glycogen synthase kinase 3 beta (GSK3β) at tyrosine 216,^37^ a modification known to reduce the kinase activity of GSK3β.^38^ Notably, inhibition of GSK3β activity, which leads to increased intracellular β-catenin levels, has been shown to promote the differentiation of dental pulp stem cells into odontoblasts.^39^ Thus, we speculate that PTPRD may promote odontoblast differentiation by dephosphorylating GSK3β at tyrosine 216, which result in cytoplasmic accumulation of β-catenin, activation of Wnt/β-catenin signaling, and upregulation of differentiation-related genes such as *Dmp1* and *Dspp*. Moreover, double knockdown of *March2* and *Ptprd* remarkably reversed the facilitating effects by knockdown of *March2* alone, indicating that the inhibitory role of MARCH2 in odontoblastic differentiation was mainly mediated through the ubiquitination and subsequent degradation of PTPRD.

**Fig. 8:**
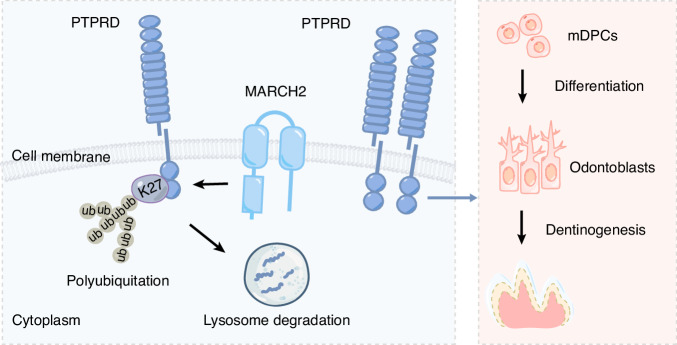
A scheme shows that MARCH2 suppresses odontoblast differentiation by promoting the K27-linked polyubiquitination and subsequent degradation of a membrane protein PTPRD, which plays a positive role in odontoblast differentiation

Among all E3 ubiquitin ligases involved in tooth development, SMURF1 was the first to be identified.^20^ In recent years, research on E3 ubiquitin ligases in tooth development has increased considerably. In the nucleus, MDM2 and WWP2 monoubquitinate distal-less homeobox 3 (DLX3) and kruppel-like factor 5 (KLF5), respectively, enhancing their transcriptional regulatory activities on the *Dmp1* and *Dspp* genes.^21,23^ Additionally, WWP2 promotes phosphatase and tensin homolog (PTEN) degradation through polyubiquitination, thereby relieving its inhibitory effect on KLF5.^24^ Conditional knockout of *Mdm2* in the odontoblast layer or global knockout of *Wwp2* in mice results in molars with thinner dentin and shorter roots.^22,24^ Another E3 ubiquitin ligase, CHIP promote the polyubiquitination and subsequent degradation of DLX3, thus inhibiting odontoblast differentiation. Notably, *Chip*-deficient mice exhibit increased dentin formation.^25^ Our study shows that MARCH2 suppresses odontoblast differentiation and dentin formation. Mechanistically, PTPRD, a membrane protein, is able to promote the odontoblastic differentiation of mDPCs. MARCH2 suppresses odontoblast differentiation through mediating the K27-linked polyubiquitination and subsequent lysosomal degradation of PTPRD (Fig. 8).

Our findings demonstrate an important mechanism by which the differentiation of odontoblasts is delicately regulated at the membrane protein level through ubiquitination modification. Given that MARCH2 destabilizes PTPRD and consequently suppresses odontoblast differentiation, modulation of this axis may represent a promising strategy for dentin regeneration. For instance, pharmacological inhibition of MARCH2 activity or stabilization of PTPRD could potentially enhance odontoblast differentiation, offering therapeutic benefit in conditions such as dental pulp injury or dentin caries. Future studies are warranted to explore the feasibility and safety of targeting the MARCH2-PTPRD axis in dentin tissue engineering and regenerative dentin therapies.

## Materials and methods

### Animals

All animal experiments were performed following the guidelines of the Animal Ethics Committee of Wuhan University (BZ20230063). *March2* deficient (*March2* KO) mice were kindly provided by Professor Hong-Bing Shu from Wuhan University. All mice were housed in a specific pathogen-free (SPF) animal facility at the Center for Animal Experiment, Wuhan University. Housing conditions were strictly controlled, with a temperature of 20–24 °C, humidity of 40%–70%, and a 12-h light/dark cycle. No more than four mice were kept per cage. Comprehensive information on the experimental mice is shown in Supplementary Table 1.

### Genotyping and tissue preparation

*March2* heterozygous mice were interbred, and the offspring underwent toe amputation on PN7. The toe tissue was subjected to heating in 200 μL 50 mol/L NaOH for 30 min (min), followed by adding 20 μL 1 mol/L Tris-HCl (pH 8.0) (Beyotime). Genotyping was performed using Polymerase Chain Reaction (PCR) analysis with primers as below: forward, 5′-GGACGTCTGGAGCTGGGCTG-3′; reverse, 5′-AGGGAGGGGCTTGCAGACCT-3′ (*March2*). The target gene sequences were amplified by PCR using 2× Hieff PCR Master Mix (Yeasen). The resulting products were separated using 1% agarose gel through electrophoresis. Then the gel was excised under ultraviolet light for subsequent Sanger sequencing.

The mandibles of *March2* deficient and littermate control mice were harvested and fixed in 4% paraformaldehyde (PFA) at 4 °C overnight, which were also applied to Kunming mice at different ages.

### Construction of AAV vectors and in vivo injection

A previous study has reported that AAV6 exhibits strong tropism for odontoblast-like cells (OLCs).^1^ We constructed an AAV6 vector expressing a shRNA targeting *March2* under the control of a mouse *Dmp1* promoter, which consists of 2 600 base pairs upstream of the transcription start site (TSS) and the first exon.^40^ A GFP reporter was included to enable tracking of transduction efficiency. The shRNA sequences are listed in Supplementary Table 2. All AAVs were obtained from Tsingke Biotechnology (Wuhan, China)

In vivo injections were performed using a 10 μL microsyringe with a 32-gauge needle via the submandibular approach as previously reported.^1^ 2 μL of AAV6-sh*March2* (2 × 10^12^ vg/mL) was injected into wild-type C57BL/6 mice at PN1; littermate controls received 2 μL of AAV6-shScr (2 × 10^12^ vg/mL) under the same conditions. To verify the in vivo transduction efficacy of AAV6-sh*March2*, mandibles were collected at PN3. To investigate the functional impact of *March2* knockdown in odontoblasts on dentin formation, mandibles were harvested at PN10, PN14 and PN21.

### Cell culture and transfection

To obtain mDPCs, mandibles were harvested from mice at E16.5 and the tooth germs were isolated under a stereomicroscope (Nikon). The dental mesenchyme was collected and digested with 0.25% trypsin (Hyclone) before being cultured in plates. The mDPCs, HEK293T cells, and Henrietta Lacks (Hela) cells were cultured in Dulbecco’s Modified Eagle’s Medium (DMEM; HyClone) containing 10% fetal bovine serum (FBS; Gibco) and 100 µ/mL penicillin/streptomycin (Hyclone). The cells were cultured in a humidified incubator at 37 °C with 5% CO_2_. mDPCs were cultured in differentiation medium (DM) with 10 mmol/L β-glycerophosphate, 50 µg/mL ascorbic acid, and 100 mol/L dexamethasone to induce odontoblastic differentiation.

siRNAs utilized for in vitro knockdown experiments were obtained from GenePharma and siRNA sequences are listed in the Supplementary Table 2. *pCMV-HA-Ub* plasmids were obtained from Miaoling. *pLOV-CMV-March2-Flag*; *pcDNA3.1-March2-myc, C64S, C67S*, and *C80S*; *pRK-HA-K6R, K11R, K27R, K29R, K33R, K48R* and *K63R*; *pRK-HA-K6O, K11O, K27O, K29O, K33O, K48O* and *K63O* were generously provided by Professor Hong-Bing Shu from Wuhan University. *pcDNA3.1-Ptprd* were provided by BaoLi Wang from Tianjin Medical University. *pcDNA3.1-Ptprd-Flag* was constructed using the Mut Express II Fast Mutagenesis Kit V2 (Vazyme). The aforementioned siRNAs and plasmids were transfected into mDPCs using the Lipofectamine 3000 Transfection Reagent (Invitrogen) following the manufacturer’s protocol, while the plasmids were transfected into HEK293T cells and Hela cells using the Lipo8000 Transfection Reagent (Beyotime) following the manufacturer’s protocol.

### H&E staining, IHC, IF, and ICC

The mandibles were decalcified by 10% Ethylene Diamine Tetraacetic Acid (EDTA), dehydrated, and embedded in paraffin. Sections at 4-µm thickness were prepared. For frozen sectioning, mandibles were fixed in 4% paraformaldehyde. Following decalcification, the samples were infiltrated sequentially with 15% and 30% sucrose at 4 °C, embedded in optimal cutting temperature (OCT) compound (Tissue-Tek, Japan), and cryosectioned at a thickness of 10 µm using a CM1950 cryostat (Leica Biosciences, Germany).

For H&E staining, tissue slides were dewaxed, rehydrated through graded alcohols, and stained with hematoxylin and eosin.

For IHC, after dewaxing and rehydration, tissue slides underwent antigen retrieval using an enzyme digestion solution (Maxin). The slides were blocked with normal serum and incubated with primary antibodies at 4 °C overnight, as listed in Supplementary Table 3. In the case of IHC, subsequent steps were performed according to the immunohistochemistry kit (Maxin) protocol. Visualization was achieved using Diaminobenzidine (DAB; Maxin), and the nuclei were counterstained with hematoxylin before mounting with neutral gum. For tissue IF, the samples were incubated with fluorescent secondary antibodies (Abbkine) at 37 °C for 1 h (h), counterstained using DAPI-containing mounting medium (ZSGB-Bio), and finally visualized under a Leica DM4000B microscope (Leica).

For ICC, mDPCs and Hela cells were cultured on cover glasses (Nest). To label intracellular lysosomes, Hela cells were treated with 200 nmol/L LysoTracker Red DND-99 (Thermo Fisher) for 1 h before sample collection. Next, the cells were fixed with 4% PFA, permeabilized using 0.1% Triton X-100, blocked with 2.5% BSA, and incubated with the primary antibody at 4 °C overnight. Subsequent steps were carried out the same as those for tissue IF. The images were captured using the FluoView FV1200 (Olympus).

### ALP staining and ARS staining

For ALP staining, mDPCs were cultured with growth medium (GM) or DM for 4 days (d) and then stained with the BCIP/NBT Alkaline Phosphatase Kit (Beyotime) according to the manufacturer’s instruction.

For ARS staining, mDPCs were cultured with either GM or DM for 5 d, then fixed and stained with 1% Alizarin Red S (Alladin). The stained cells were then eluted at 37 °C overnight with 10% cetylpyridinium chloride (CPC), and the absorbance of the eluate at 562 nm was measured for quantification.

### RT-qPCR analysis

Total RNAs were extracted from cells using Trizol reagent (Takara), and cDNAs were synthesized from 1 μg RNAs with the ABScript III RT Master Mix (Abclonal). The reaction system was prepared according to the instruction provided with the ChamQ SYBR qPCR Master Mix (Yeason) for RT-qPCR program. PCR primers are listed in the Supplementary Table 2.

### WB analysis

Cells were lysed using NP40 (Beyotime) and Cocktail (MedChemExpress), and the lysate was then sonicated. After centrifugation, the protein supernatant was collected and mixed with 5× SDS-PAGE Sample Loading Buffer (Biosharp), then heated at 95 °C. Protein separation was performed by SDS-PAGE gel, followed by transfer to a polyvinylidene difluoride membrane (Millipore). The membrane was incubated with 5% non-fat milk in TBS containing 0.1% Tween 20 (Biosharp) at room temperature for 1 h, then with primary antibodies at 4 °C overnight, as listed in Supplementary Table 3. Afterwards, the membrane was incubated with secondary antibodies for 1 h at room temperature, followed by chemiluminescent detection using Western Bright ECL (Advansta) and Super ECL Detection Reagent (Yeasen). Protein bands were visualized using Dual-color near-infrared fluorescence imaging system (LI-COR). ImageJ was used to quantify band intensities.

### Co-IP assay

Cells were lysed with NP40 and Cocktail, and then sonicated. After centrifugation, the supernatant was collected. Ten percent of the supernatant was reserved as the input, and 1 mg cell lysate was incubated with anti-FLAG antibody, anti-MYC antibody, or nonimmune IgG under rotation at 4 °C overnight. Subsequently, 40 µL of Protein A/G magnetic beads (Bimake) were added and incubated at 4 °C for 1 h under rotation. The beads were next washed for five times with lysis buffer and resuspended in 80 µL of 1× SDS buffer, and heated at 95 °C. The samples were then analyzed by WB analysis.

### Micro-CT

Micro-CT scanning was performed using a SkyScan 1276 (Bruker) with an effective pixel size of 10 μm, at 50 kV voltage, 114 μA current, and a 14 min exposure time across 180 rotational steps. The two-dimensional image slices were reconstructed using the CTAn software, and quantitative analyses were conducted with Mimics Research 21.0.

### Double-Calcein labeling

*March2* deficient mice and control littermates at PN5 were intraperitoneally injected with 20 mg/kg Calcein (2.5 mg/mL in 2% NaHCO_3_ solution) (Sigma) with a second injection at PN10. At PN14, the mice were euthanized, and the mandibles were collected. After fixation and washes, the samples were sent to Servicebio company for tissue dehydration, embedding, and sectioning. Slices were observed under the Aperio digital pathology (Leica).

### In situ PLA assay

The detailed procedure for in situ PLA has been previously reported.^41,42^ Briefly, cells on cover glasses were treated with 0.1% Triton X-100, and then blocked using reagents from the Duolink In Situ PLA Probe (Sigma). Then the cells were incubated with primary antibodies at 4 °C overnight. Next, the PLA probe solution was applied and incubated at 37 °C for 1 h. Amplification process was completed using the Duolink In Situ Red Detection Reagents (Sigma). The slices were subsequently mounted with DAPI-containing mounting medium and imaged using the FluoView FV1200.

### Bioinformatics analysis

The PN3.5 molar single-cell sequencing data used for single-cell analysis were retrieved from the GEO database (No. GSE189381).^13^ The single-cell data underwent quality control using the Seurat 4 and were normalized accordingly. Umap was employed for dimensionality reduction, followed by unsupervised clustering based on gene expression patterns. Classical marker genes were utilized to identify different cell subpopulations.

### Statistical analysis

All experiments were conducted independently at least three times. Statistical analyses were carried out using GraphPad Prism software (version 8.0). Normality of the data was assessed using the Shapiro–Wilk test, and homogeneity of variances was evaluated with Levene’s test. The differences between two groups were analyzed by using Student’s *t*-test, and three or more groups were analyzed by one-way ANOVA with Tukey’s post hoc test or Dunnett’s post hoc test, with *P*-value < 0.05 considered statistically significant (^*^P < 0.05; ^**^P < 0.01; ^***^P < 0.001; ^****^P < 0.000 1).

## Supplementary information

Supplementary Material -- MARCH2 suppresses odontoblast differentiation by polyubiquitinating PTPRD

Raw western blot data

## References

[1] Fu, J. et al. AAV6-mediated gene therapy prevents developmental dentin defects in a dentinogenesis imperfecta type III mouse model. *Hum. Gene Ther.* **34**, 567–577 (2023). 37014084 10.1089/hum.2023.008

[2] Jelokhani-Niaraki, M. Membrane proteins: structure, function and motion. *Int. J. Mol. Sci.* **24**, 5 (2023). 10.3390/ijms24010468 PMC9820270 36613912

[3] Cornejo, F., Franchini, N., Cortes, B. I., Elgueta, D. & Cancino, G. I. Neural conditional ablation of the protein tyrosine phosphatase receptor Delta PTPRD impairs gliogenesis in the developing mouse brain cortex. *Front. Cell. Dev. Biol.* **12**, 13 (2024). 10.3389/fcell.2024.1357862 PMC10937347 38487272

[4] Yue, R., Zhou, B. O., Shimada, I. S., Zhao, Z. Y. & Morrison, S. J. Leptin receptor promotes adipogenesis and reduces osteogenesis by regulating mesenchymal stromal cells in adult bone marrow. *Cell Stem Cell* **18**, 782–796 (2016). 27053299 10.1016/j.stem.2016.02.015

[5] Fu, Y. et al. Identification of GPI-anchored protein LYPD1 as an essential factor for odontoblast differentiation in tooth development. *J. Biol. Chem.* **299**, 17 (2023). 10.1016/j.jbc.2023.104638 PMC10130355 36963497

[6] Patwardhan, A., Cheng, N. & Trejo, J. Post-translational modifications of G protein-coupled receptors control cellular signaling dynamics in space and time. *Pharmacol. Rev.* **73**, 120–151 (2021). 33268549 10.1124/pharmrev.120.000082 PMC7736832

[7] Reiche, J. & Huber, O. Post-translational modifications of tight junction transmembrane proteins and their direct effect on barrier function. *Biochim. Biophys. Acta Biomembr.* **1862**, 11 (2020). 10.1016/j.bbamem.2020.183330 32376223

[8] Lee, H. et al. Head formation requires Dishevelled degradation that is mediated by March2 in concert with Dapper1. *Development* **145**, 13 (2018). 10.1242/dev.143107 29549110

[9] Zeng, L. W. et al. The membrane-associated ubiquitin ligases MARCH2 and MARCH3 target IL-5 receptor alpha to negatively regulate eosinophilic airway inflammation. *Cell. Mol. Immunol.* **19**, 1117–1129 (2022). 35982175 10.1038/s41423-022-00907-9 PMC9508171

[10] Liu, X. M. et al. MARCH8 inhibits influenza A virus infection by targeting viral M2 protein for ubiquitination-dependent degradation in lysosomes. *Nat. Commun.* **12**, 13 (2021). 34285233 10.1038/s41467-021-24724-2 PMC8292393

[11] Castellanos, C. A. et al. Lymph node-resident dendritic cells drive TH2 cell development involving MARCH1. *Sci. Immunol.* **6**, 17 (2021). 10.1126/sciimmunol.abh0707 PMC8736284 34652961

[12] Iyengar, P. V., Hirota, T., Hirose, S. & Nakamura, N. Membrane-associated RING-CH 10 (MARCH10 Protein) is a microtubule-associated E3 ubiquitin ligase of the spermatid flagella. *J. Biol. Chem.* **286**, 39082–39090 (2011). 21937444 10.1074/jbc.M111.256875 PMC3234733

[13] Jing, J. J. et al. Spatiotemporal single-cell regulatory atlas reveals neural crest lineage diversification and cellular function during tooth morphogenesis. *Nat. Commun.* **13**, 14 (2022). 35974052 10.1038/s41467-022-32490-y PMC9381504

[14] Lin, H., Li, S. & Shu, H. B. The membrane-associated MARCH E3 ligase family: emerging roles in immune regulation. *Front. Immunol.* **10**, 10 (2019). 31404274 10.3389/fimmu.2019.01751 PMC6669941

[15] Tomita, H. et al. The protein tyrosine phosphatase receptor delta regulates developmental neurogenesis. *Cell Rep.* **30**, 215 (2020). 31914388 10.1016/j.celrep.2019.11.033

[16] Chen, M. et al. Metastasis suppressor 1 interacts with protein tyrosine phosphatase receptor-δ to regulate adipogenesis. *Faseb J.* **37**, 13 (2023). 10.1096/fj.202201322R 36906292

[17] Liu, Y. C., Penninger, J. & Karin, M. Immunity by ubiquitylation: a reversible process of modification. *Nat. Rev. Immunol.* **5**, 941–952 (2005). 16322747 10.1038/nri1731 PMC7096784

[18] Cheng, J. Y. et al. Linear polyubiquitylation of Gli protein regulates its protein stability and facilitates tumor growth in colorectal cancer. *Cell Death Discov.* **10**, 14 (2024). 39164252 10.1038/s41420-024-02147-4 PMC11335874

[19] Rape, M. Ubiquitylation at the crossroads of development and disease. *Nat. Rev. Mol. Cell Biol.* **19**, 59–70 (2018). 28928488 10.1038/nrm.2017.83

[20] Lee, D. S. et al. Crosstalk between nuclear factor I-C and transforming growth factor-β1 signaling regulates odontoblast differentiation and homeostasis. *PLoS ONE* **6**, 12 (2011). 10.1371/journal.pone.0029160 PMC3241690 22195013

[21] Zheng, H., Yang, G., Fu, J., Chen, Z. & Yuan, G. Mdm2 promotes odontoblast-like differentiation by ubiquitinating Dlx3 and p53. *J. Dent. Res.* **99**, 320–328 (2020). 31847675 10.1177/0022034519893672

[22] Zheng, H., Fu, J., Chen, Z., Yang, G. & Yuan, G. Dlx3 ubiquitination by nuclear Mdm2 is essential for dentinogenesis in mice. *J. Dent. Res.* **101**, 1064–1074 (2022). 35220830 10.1177/00220345221077202

[23] Fu, J. et al. WWP2 promotes odontoblastic differentiation by monoubiquitinating KLF5. *J. Dent. Res.* **100**, 432–439 (2021). 33164644 10.1177/0022034520970866

[24] Fu, J. et al. A WWP2-PTEN-KLF5 signaling axis regulates odontoblast differentiation and dentinogenesis in mice. *J. Biol. Chem.* **298**, 12 (2022). 10.1016/j.jbc.2022.102220 PMC9358474 35780838

[25] Zheng, H. W. et al. CHIP inhibits odontoblast differentiation through promoting DLX3 polyubiquitylation and degradation. *Development* **150**, 13 (2023). 10.1242/dev.200848 37213079

[26] Nakamura, N., Fukuda, H., Kato, A. & Hirose, S. MARCH-II is a syntaxin-6-binding protein involved in endosomal trafficking. *Mol. Biol. Cell* **16**, 1696–1710 (2005). 15689499 10.1091/mbc.E04-03-0216 PMC1073653

[27] Yoo, W., Cho, E. B., Kim, S. & Yoon, J. B. The E3 ubiquitin ligase MARCH2 regulates ERGIC3-dependent trafficking of secretory proteins. *J. Biol. Chem.* **294**, 10900–10912 (2019). 31142615 10.1074/jbc.RA119.007435 PMC6635435

[28] Xia, D. et al. MARCH2 regulates autophagy by promoting CFTR ubiquitination and degradation and PIK3CA-AKT-MTOR signaling. *Autophagy* **12**, 1614–1630 (2016). 27308891 10.1080/15548627.2016.1192752 PMC5082784

[29] Li, H. L. et al. Linc00707 regulates autophagy and promotes the progression of triple negative breast cancer by activation of PI3K/AKT/mTOR pathway. *Cell Death Discov*. **10**, 10.1038/s41420-024-01906-7 (2024) 10.1038/s41420-024-01906-7 PMC10940671 38485945

[30] Liu, S. L. et al. The E3 ubiquitin ligase MARCH2 protects against myocardial ischemia-reperfusion injury through inhibiting pyroptosis via negative regulation of PGAM5/MAVS/NLRP3 axis. *Cell Discov.* **10**, 23 (2024). 38409220 10.1038/s41421-023-00622-3 PMC10897310

[31] Shimazu, J., Wei, J. W. & Karsenty, G. Smurf1 inhibits osteoblast differentiation, bone formation, and glucose homeostasis through serine 148. *Cell Rep.* **15**, 27–35 (2016). 27052174 10.1016/j.celrep.2016.03.003 PMC4826790

[32] Dikic, I. *Annual Review of Biochemistry*, Vol. 86 (ed Kornberg, R. D.) 193–224 (Annual Reviews, 2017). 10.1146/annurev-biochem-061516-044908 28460188

[33] Chmilewsky, F. et al. C5L2 regulates DMP1 expression during odontoblastic differentiation. *J. Dent. Res.* **98**, 597–604 (2019). 30702959 10.1177/0022034518820461 PMC6481006

[34] Miyazaki, A. et al. Coordination of WNT signaling and ciliogenesis during odontogenesis by piezo type mechanosensitive ion channel component 1. *Sci. Rep.* **9**, 13 (2019). 31611621 10.1038/s41598-019-51381-9 PMC6791893

[35] Mohamed, F. F., Ge, C., Binrayes, A. & Franceschi, R. T. The role of discoidin domain receptor 2 in tooth development. *J. Dent. Res.* **99**, 214–222 (2020). 31869264 10.1177/0022034519892563 PMC7315682

[36] Cornejo, F., Cortes, B. I., Findlay, G. M. & Cancino, G. I. LAR Receptor tyrosine phosphatase family in healthy and diseased brain. *Front. Cell. Dev. Biol.* **9**, 21 (2021). 10.3389/fcell.2021.659951 PMC8711739 34966732

[37] Henderson, I. M. et al. Substrate-selective positive allosteric modulation of PTPRD’s phosphatase by flavonols. *Biochem. Pharmacol.* **202**, 10.1016/j.bcp.2022.115109 (2022). 10.1016/j.bcp.2022.115109 PMC10184881 35636503

[38] Shimasaki, T. et al. Glycogen synthase kinase 3β inhibition sensitizes pancreatic cancer cells to gemcitabine. *J. Gastroenterol.* **47**, 321–333 (2012). 22041920 10.1007/s00535-011-0484-9

[39] Rajasekar, V., Abdalla, M. M., Basbrain, M. S., Neelakantan, P. & Yiu, C. K. Y. Odontogenic differentiation of dental pulp stem cells by glycogen synthase kinase-3β inhibitory peptides. *Stem Cell Res. Therapy* **16**, 10.1186/s13287-025-04150-7 (2025). 10.1186/s13287-025-04150-7 PMC11792195 39901291

[40] Chen, Z., Xie, H., Yuan, J., Lan, Y. & Xie, Z. Kruppel-like factor 6 promotes odontoblastic differentiation through regulating the expression of dentine sialophosphoprotein and dentine matrix protein 1 genes. *Int. Endod. J.* **54**, 572–584 (2021). 33200415 10.1111/iej.13447

[41] Bagchi, S., Fredriksson, R. & Wallen-Mackenzie, A. In situ proximity ligation assay (PLA). *Methods Mol. Biol.* **1318**, 149–159 (2015). 26160573 10.1007/978-1-4939-2742-5_15

[42] Xu, X. M. et al. Dentin sialoprotein acts as an angiogenic factor through association with the membrane receptor endoglin. *J. Biol. Chem*. **301**, 10.1016/j.jbc.2025.108279 (2025) 10.1016/j.jbc.2025.108279 PMC11910139 39922489

