# Supplementary material for: MARCH2 suppresses odontoblast differentiation by polyubiquitinating PTPRD
Source: Int J Oral Sci. 2026 Jan 10;18:5. doi: 10.1038/s41368-025-00407-2 (PMC12789588; doi:10.1038/s41368-025-00407-2)
Supplement: Supplementary file 1 — Supplementary Material -- MARCH2 suppresses odontoblast differentiation by polyubiquitinating PTPRD [file 41368_2025_407_MOESM1_ESM.docx]

**TITLE: MARCH2 suppresses odontoblast differentiation by polyubiquitinating PTPRD**

**RUNNING TITLE: MARCH2 suppresses odontoblast differentiation**

**AUTHORS：**

Hao Feng^1,2,3^, Jiaxin Niu^1,2,3^, Zhi Chen^1^, Guobin Yang^1^, Guohua Yuan^1,2,3^**^*^**

**AFFILIATION:**

^1^State Key Laboratory of Oral & Maxillofacial Reconstruction and Regeneration, Key Laboratory of Oral Biomedicine Ministry of Education, Hubei Key Laboratory of Stomatology, School & Hospital of Stomatology, Wuhan University, Wuhan 430079, China.

^2^Frontier Science Center for Immunology and Metabolism, Wuhan University, Wuhan 430079, China.

^3^Hubei Provincial Key Laboratory of Developmentally Originated Disease, Wuhan University, Wuhan 430071, China.

*Corresponding author: Guohua Yuan ([yuanguohua@whu.edu.cn](mailto:yuanguohua@whu.edu.cn))

**SUPPLEMENTARY INFORMATION**

**SUPPLEMENTARY TABLES**

**Supplementary Table 1**. Animal samples details.

The *March2* deficient and littermate control mice were of C57BL/6 strain, and all postnatal mice used were male. The genotypes and ages of the mice used in this study are specified in the Supplementary Table 1. The time points for tissue collection in mice effectively encompass the period of odontoblast differentiation and dentinogenesis.^1^ The number of mice used in this study was determined based on the sample sizes reported in previous literature.^2^ Researchers were aware of group allocation at all stages of the study, including during allocation, conduct, outcome assessment, and data analysis. Dental papilla cells (DPCs) were isolated from Kunming mice. There are no important adverse events in each experimental group. We made every effort to minimize the pain and stress that animals suffered in this study.

| Genotype | Age | Experiments | Sample number  (n=) | Total number |
| --- | --- | --- | --- | --- |
| *March2^+/+^*  (Control littermates) | PN7 | Histological analysis | 3 | 3 |
|  | PN10 | Micro-CT, histological analysis | 5 | 5 |
|  | PN14 | Micro-CT, histological and Calcein labeling analysis | 10 | 10 |
| *March2^-/-^*  (*March2* Deficient) | PN7 | Histological analysis | 3 | 3 |
|  | PN10 | Micro-CT, histological analysis | 5 | 5 |
|  | PN14 | Micro-CT, histological and Calcein labeling analysis | 10 | 10 |
| Wild-type C57BL/6 mice | PN1 | AAV injection | 90 | 90 |
| Kunming mice | E16.5 | Primary mDPCs isolation | 400 | 400 |
| Kunming mice | E18.5,  PN2,  PN3, PN7, PN10 | Histological analysis | 3 for each age | 15 |

**Supplementary Table 2**. The sequences of primers, siRNAs, and shRNAs used in this study.

| Gene | Primer or siRNA Sequences |
| --- | --- |
| *mDmp1* | forward: 5’-ACCACAATACTGAATCTGAAAGCTC-3’ |
|  | reverse: 5’-TGCTGTCCGTGTGGTCACTA-3’ |
| *mDspp* | forward: 5’-TAGCACCAACCATGAGGCTG-3’ |
|  | reverse: 5’-TGTTGCCTTTGTTGGGACCT-3’ |
| *mCol1a1* | forward: 5’-TTCGTGACCGTGACCTTGAG-3’ |
|  | reverse: 5’-CGATCTCGTTGGATCCCTGG-3’ |
| *mPtprd* | forward: 5’-AATGGACCCCACTCAGCATG-3’ |
|  | reverse: 5’-ATCTCCCAAGACAGCAGAGCAC-3’ |
| *mGAPDH* | forward: 5’-TGTGTCCGTCGTGGATCTGA-3’ |
|  | reverse: 5’-TTGCTGTTGAAGTCGCAGGAG-3’ |
| *mMarch1* | forward: 5’-AAAACTGGTTGTGGTGGCTATT-3’ |
|  | reverse: 5’-ACAAAGATCACACGGTTGTAGG-3’ |
| *mMarch2* | forward: 5’-GCCCTCTTTACCATCTATGTGC-3’ |
|  | reverse: 5’-TCAGGCGGACTTTCTGATTTG-3’ |
| *mMarch3* | forward: 5’-TGAAGACGGTGGAGGATTGTG-3’ |
|  | reverse: 5’-GAGTCCGCACTACTGTTGACA-3’ |
| *mMarch4* | forward: 5’-GCCGCTGTCGAATGCTCTT-3’ |
|  | reverse: 5’-GCCGGAAGGGTGTTGTTGT-3’ |
| *mMarch5* | forward: 5’-GACCAAGCCCTTCAACAGATG-3’ |
|  | reverse: 5’-TGGTGAACCCACTTAGTAGATCC-3’ |
| *mMarch6* | forward: 5’-CTCAGTGGTGGTTGAAAACTGT-3’ |
|  | reverse: 5’-GCGCCAGAAGCTATGACATAC-3’ |
| *mMarch7* | forward: 5’-TTGCTGTCACGGATAGCTTCT-3’ |
|  | reverse: 5’-GGTTCCATTATTCCGAGACTGTG-3’ |
| *mMarch8* | forward: 5’-TCTCTCGCACTTCTGTCACAC-3’ |
|  | reverse: 5’-GCAAGCCTGATGCACGAAATG-3’ |
| *mMarch9* | forward: 5’-CGCTGTGGTTATACGATCCTG-3’ |
|  | reverse: 5’-ACAACCTCTCGACCAGAATGA-3’ |
| *mMarch10* | forward: 5’-CAAGATACCAGGTTACCCAGTGA-3’ |
|  | reverse: 5’-GGATAACACCATCAGATTTCGCA-3’ |
| *mMarch11* | forward: 5’-CGAAACACGCTCCGTATGTAG-3’ |
|  | reverse: 5’-GGATTTAACAACTCACCCTGCT-3’ |
| si*March2* | (1)5’-3’ CAUCCGGGCUUUGGAUUCATT |
|  | (1)3’-5’ UGAAUCCAAAGCCCGGAUGTT |
|  | (2)5’-3’ CCUGAAGAUUCGGGAAGCATT |
|  | (2)3’-5’ UGCUUCCCGAAUCUUCAGGTT |
| si*Ptprd* | (1)5’-3’ CCGGACAUUUGCACUUUAUTT |
|  | (1)3’-5’ AUAAAGUGCAAAUGUCCGGTT |
|  | (2)5’-3’ CUGGCUGCUUCAUUGUAAUTT |
|  | (2)3’-5’ AUUACAAUGAAGCAGCCAGTT |
| Scramble siRNA and shRNA | 5’-3’ UUCUCCGAACGUGUCACGUTT |
|  | 3’-5’ ACGUGACACGUUCGGAGAATT |
| sh*March2* | 5’-3’ CCUGAAGAUUCGGGAAGCATT |
|  | 3’-5’ UGCUUCCCGAAUCUUCAGGTT |

| **Supplementary Table 3**. Antibodies used in this study. | | | | | |
| --- | --- | --- | --- | --- | --- |
| **Antibody specificity** | **Species** | **Brands** | **Catalog#** | **Usages** | **Concentrations** |
| **Anti-MARCH2** | Rabbit | ImmunoWay | WT2643 | IHC ICC | 1:200 |
| **Anti-MARCH2** | Rabbit | Zenbio | 823811 | WB | 1:2000 |
| **Anti-PTPRD** | Rabbit | Proteintech | 27941-1-AP | WB | 1:1000 |
| **Anti-PTPRD** | Rabbit | Proteintech | 27941-1-AP | IHC IF ICC | 1:200 |
| **Anti-DMP1** | Rabbit | Abcolnol | A16832 | WB | 1:2000 |
| **Anti-DMP1** | Rabbit | Abcolnol | A16832 | IF | 1:200 |
| **Anti-DSPP** | Rabbit | Novus | NBP2-92546 | WB | 1:2000 |
| **Anti-DSPP** | Rabbit | Novus | NBP2-92546 | IF | 1:200 |
| **Anti-COL1A1** | Rabbit | proteintech | 14695-1-AP | WB | 1:2000 |
| **Anti-COL1A1** | Rabbit | proteintech | 14695-1-AP | IF | 1:200 |
| **Anti-β-ACTIN** | Rabbit | Abcolnol | AC038 | WB | 1:10000 |
| **Anti-FLAG** | Mouse | Abcolnol | AE005 | WB | 1:3000 |
| **Anti-HA** | Mouse | Abcolnol | AE008 | WB | 1:3000 |
| **Anti-MYC** | Mouse | proteintech | 60003-2-IG | WB | 1:3000 |
| **Anti-MYC** | Mouse | proteintech | 60003-2-IG | ICC | 1:400 |
| **Anti-MYC** | Rabbit | Abcolnol | AE092 | ICC | 1:400 |
| **Anti-Ub** | Mouse | ImmunoWay | YM3636 | ICC | 1:200 |

**SUPPLEMENTARY FIGURES AND FIGURE LEGENDS**


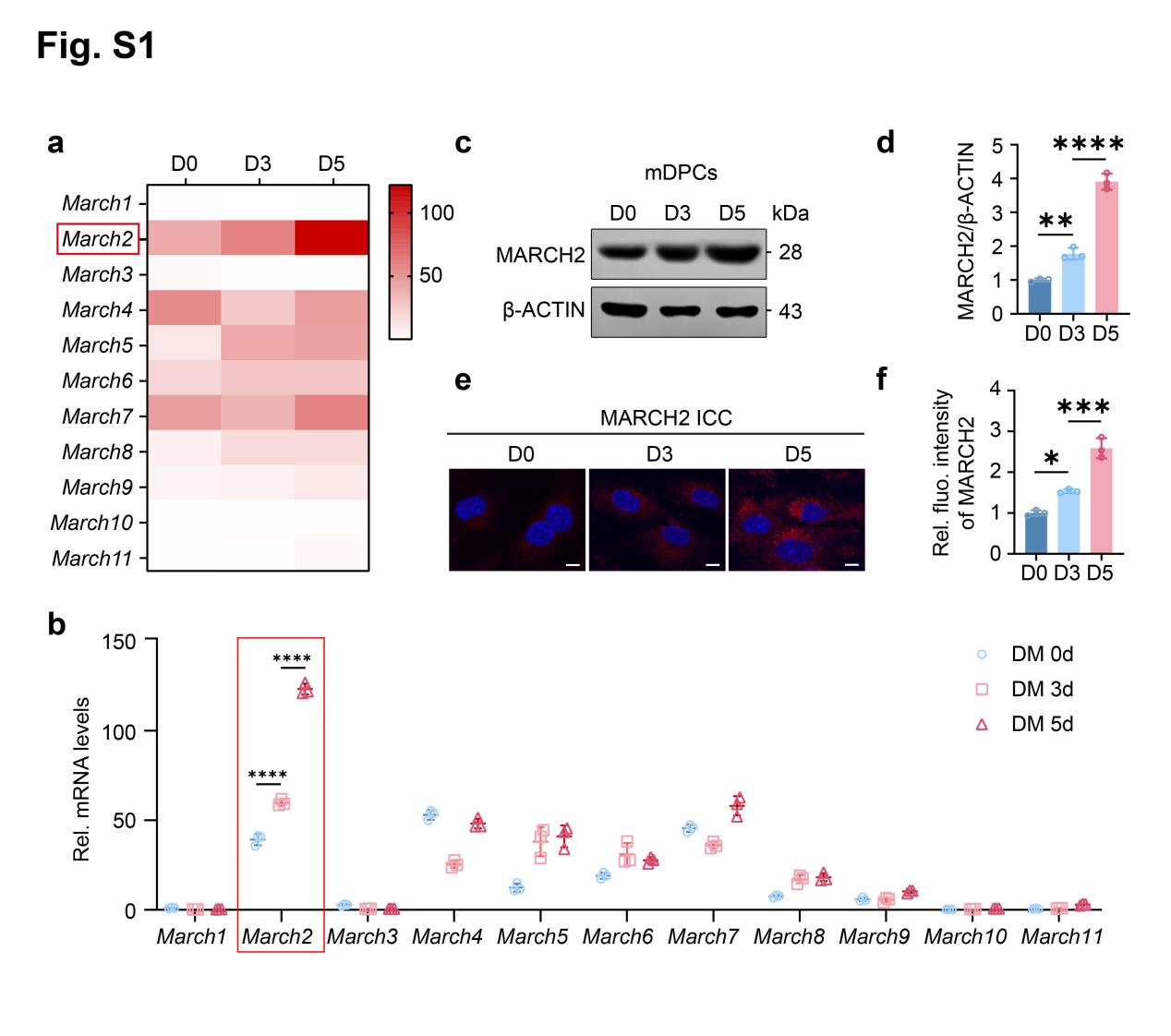


**Fig. S1** The expression pattern of MARCH2 during odontoblastic differentiation of mouse dental papilla cells (mDPCs) *in vitro.* **a-b** Heat map and dot plots show relative mRNA levels of the *March* family members in mDPCs after differentiation induction for 0 day (d), 3 d and 5 d assessed by Real-Time Quantitative PCR (RT-qPCR) (n = 3). The mRNA level of *March1* at 0 d of differentiation induction was normalized to 1. **c** The protein level of MARCH2 in mDPCs after differentiation induction for 0 d, 3 d and 5 d shown by western blot (WB) analysis. **d** Quantification of the relative expression level of MARCH2 in (**c**) (n = 3). **e** Immunocytochemistry (ICC) shows the expression pattern of MARCH2 in mDPCs after differentiation induction for 0 d, 3 d and 5 d. Scale bar, 10 μm. **f** Quantification of the relative fluorescence intensity of MARCH2 in (**e**) (n = 3). D, differentiation induction. The statistical difference was analyzed by one-way ANOVA with Tukey’s post hoc test (**b**, **d**, **f**), where **P* < 0.05; ***P* < 0.01; ****P* < 0.001; *****P* < 0.000 1.


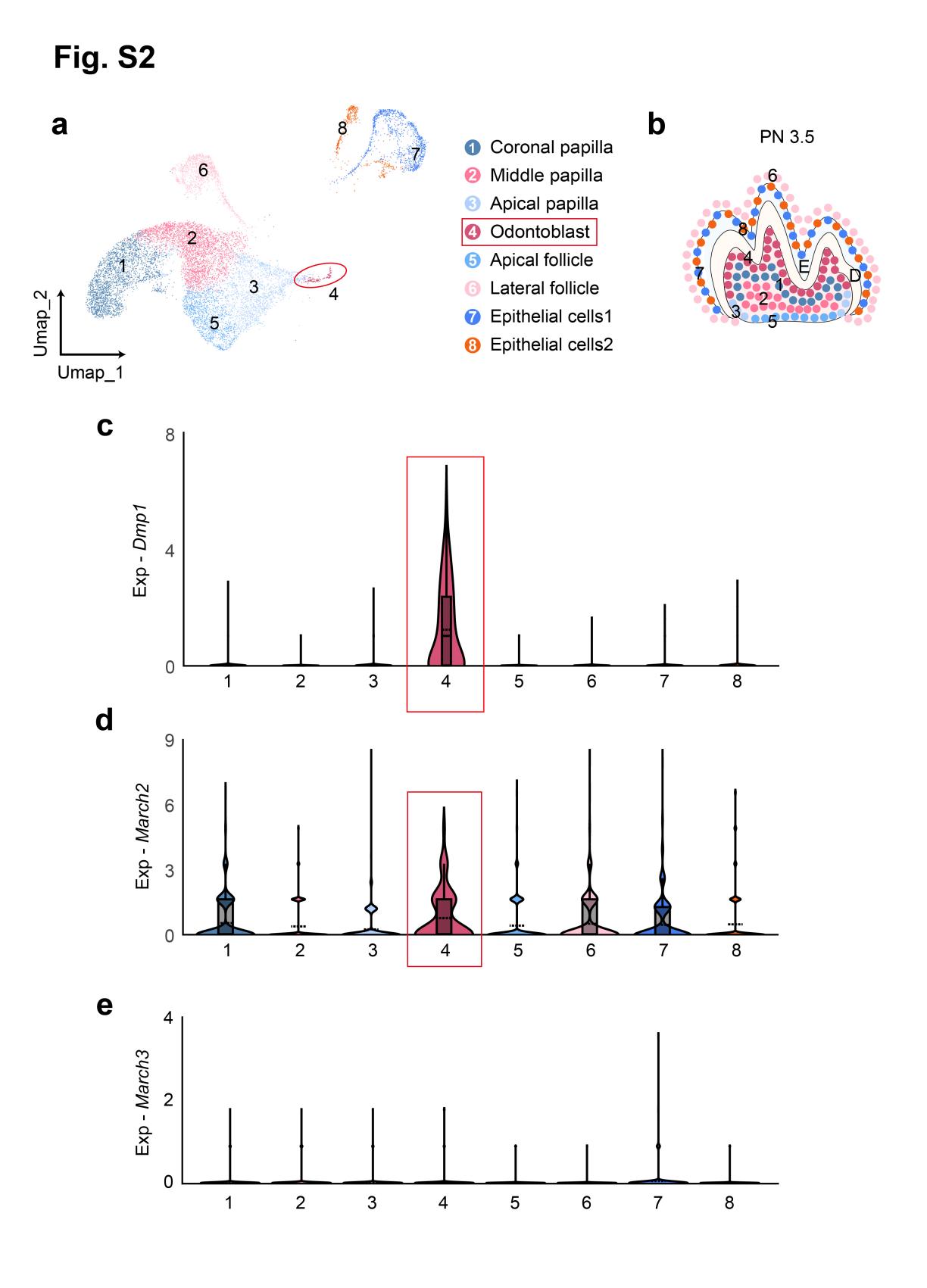


**Fig. S2** The expression of *Dmp1*, *March2* and *March3* in dental cell subpopulations. **a** Umap plot of cell clusters in mouse molars at postnatal day 3.5 (PN3.5). **b** Schematic representation of cell clustering in (**a)**. **c** The violin plot displays the expression level of *Dmp1* in the subpopulations of mouse molars at PN3.5. Notably, the cluster 4 shows specific high expression of *Dmp1*, identifying it as the odontoblast subpopulation. **d** The violin plot displays the expression level of *March2* in the subpopulations of mouse molars at PN3.5. **e** The violin plot displays the expression level of *March3* in the subpopulations of mouse molars at PN3.5.


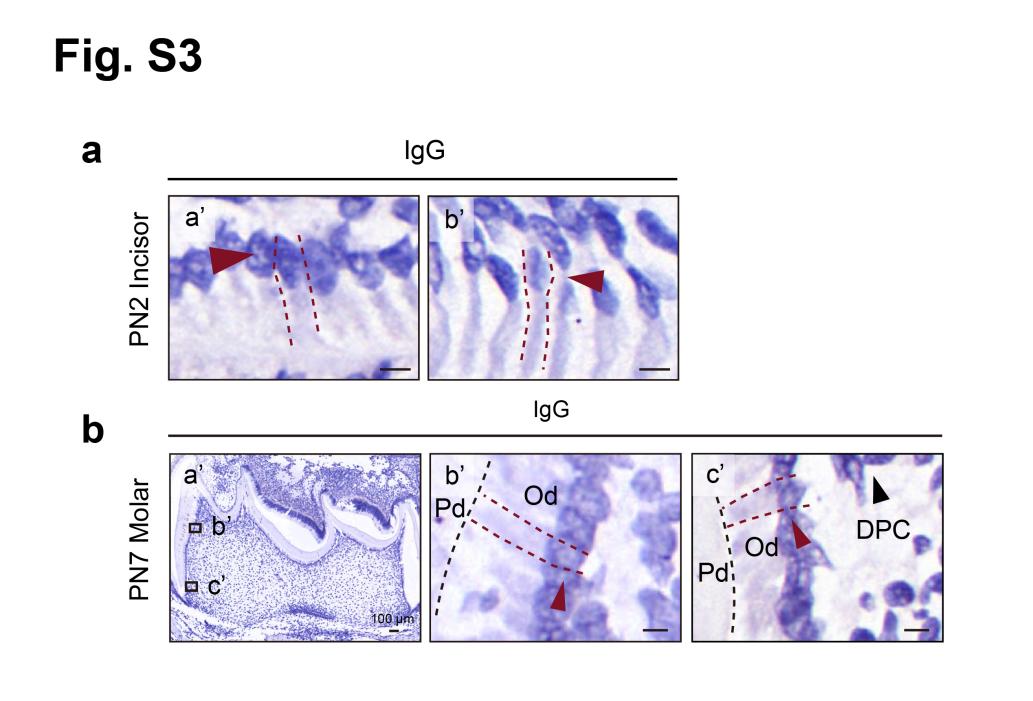


**Fig. S3** Negative control for Immunohistochemistry (IHC) using normal IgG instead of the primary antibody in PN2 mouse incisor (**a**) and PN7 mouse molars (**b**). Red arrows and red dashed lines mark the odontoblasts. Black arrows mark the dental papilla cells. The black dotted lines represent the boundaries between pre-dentin and odontoblast layer. Od, odontoblasts; DPC, dental papilla cell; Pd, pre-dentin. Scale bar, 5 μm.


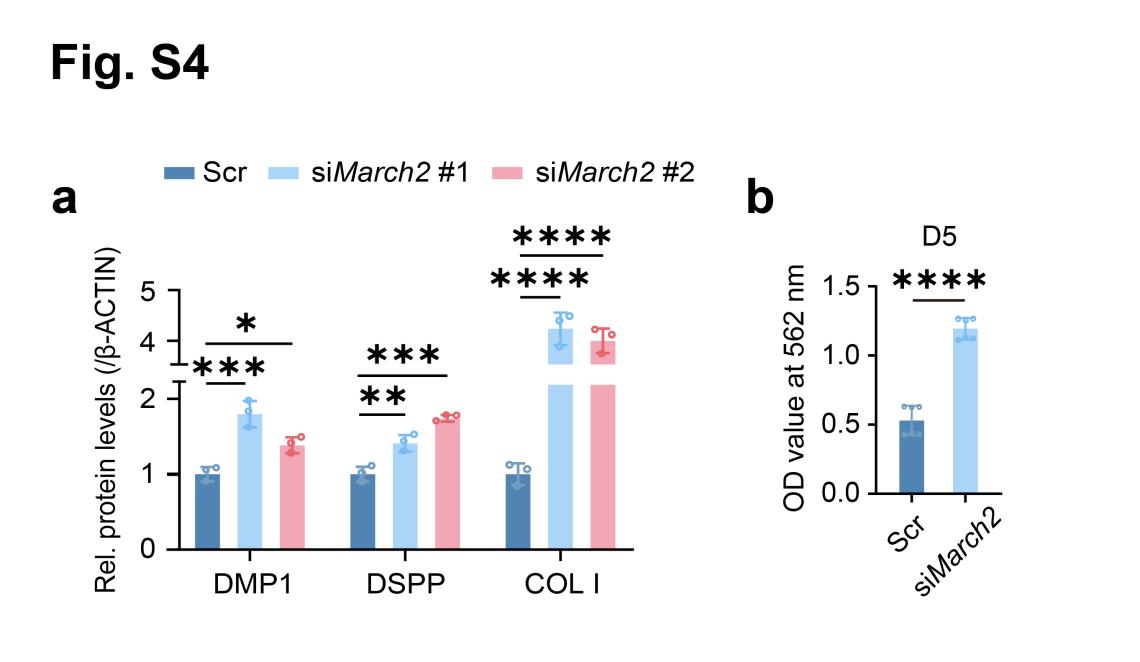


**Fig. S4** Supplementary quantification of WB analysis and Alizarin Red S (ARS) staining related to Fig. 2. **a** Quantification of the relative protein expression levels of DMP1, DSPP and COL I in Fig. 2c (n = 3). **b** For semi-quantitative analysis, stained cells shown in Fig. 2e were collected using 10% cetylpyridinium chloride (CPC), and their optical density (OD) value at 562 nm was measured (n = 6). D, differentiation induction. The statistical difference was analyzed by one-way ANOVA with Tukey’s post hoc test (**a**) and two-tailed unpaired Student’ s t-test (**b**), where **P* < 0.05; ***P* < 0.01; ****P* < 0.001; *****P* < 0.000 1.


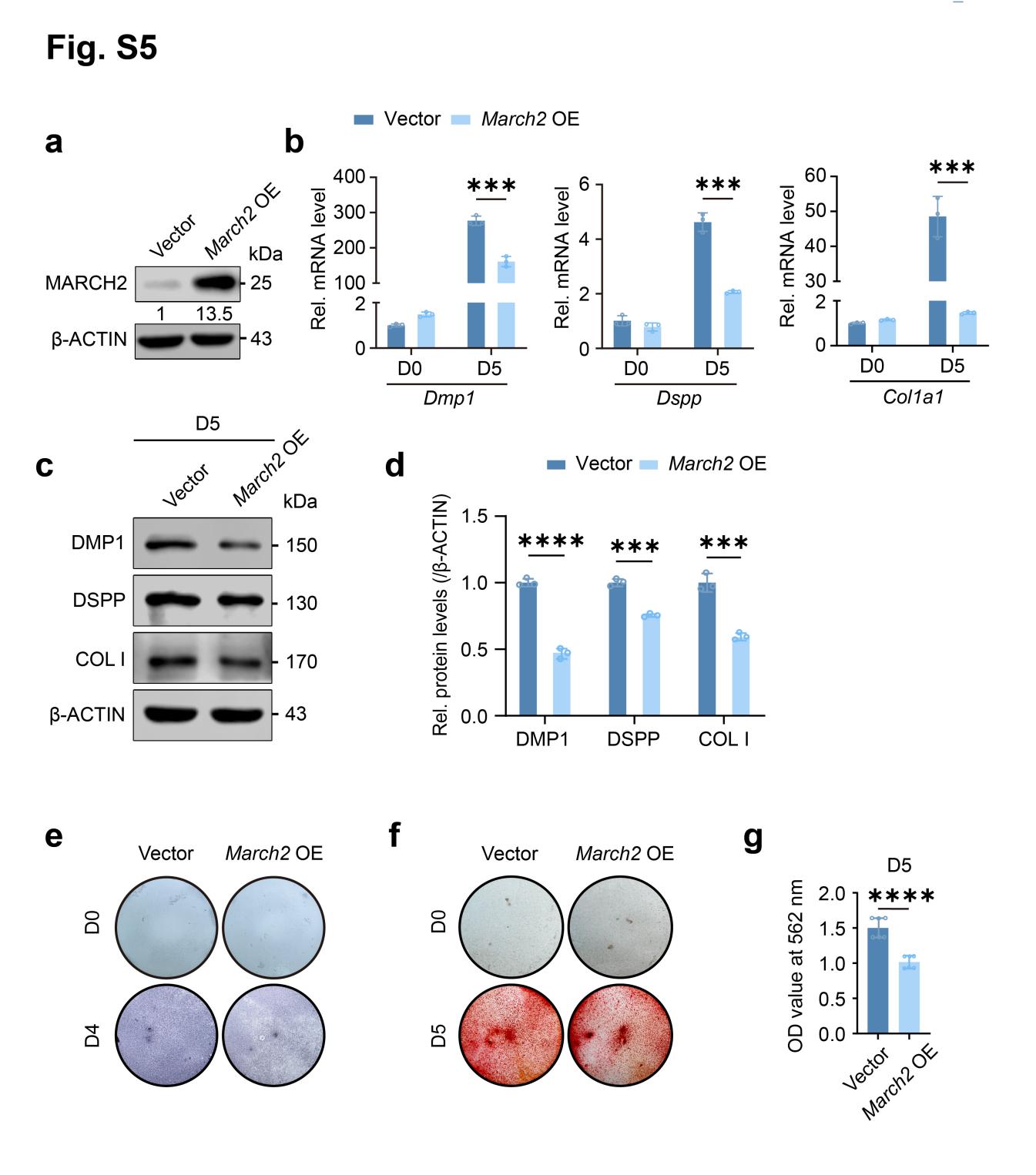


**Fig. S5** Overexpression of MARCH2 suppresses the odontoblastic differentiation of mDPCs *in vitro*. **a** The overexpressed MARCH2 in mDPCs detected by WB analysis. The ratios of gray values for MARCH2/β-ACTIN are displayed under the band. **b** The effects of MARCH2 overexpression on the mRNA levels of *Dmp1*, *Dspp* and *Col1a1* tested by RT-qPCR (n = 3). **c** The effects of MARCH2 overexpression on the protein levels of DMP1, DSPP and COL I shown by WB analysis. **d** Quantification of the relative protein expression levels of DMP1, DSPP and COL I in (**c**) (n = 3). **e** The effects of MARCH2 overexpression on the alkaline phosphatase (ALP) activity detected by ALP staining. **f** The effects of MARCH2 overexpression on the mineralized nodule formation accessed by ARS staining. **g** Semi-quantitative analysis of the OD value of the ARS-stained cells in (**f**) (n = 6). D, differentiation induction. The statistical difference was analyzed by two-tailed unpaired Student’ s t-test (**b**, **d**, **g**), where ****P* < 0.001; *****P* < 0.000 1.


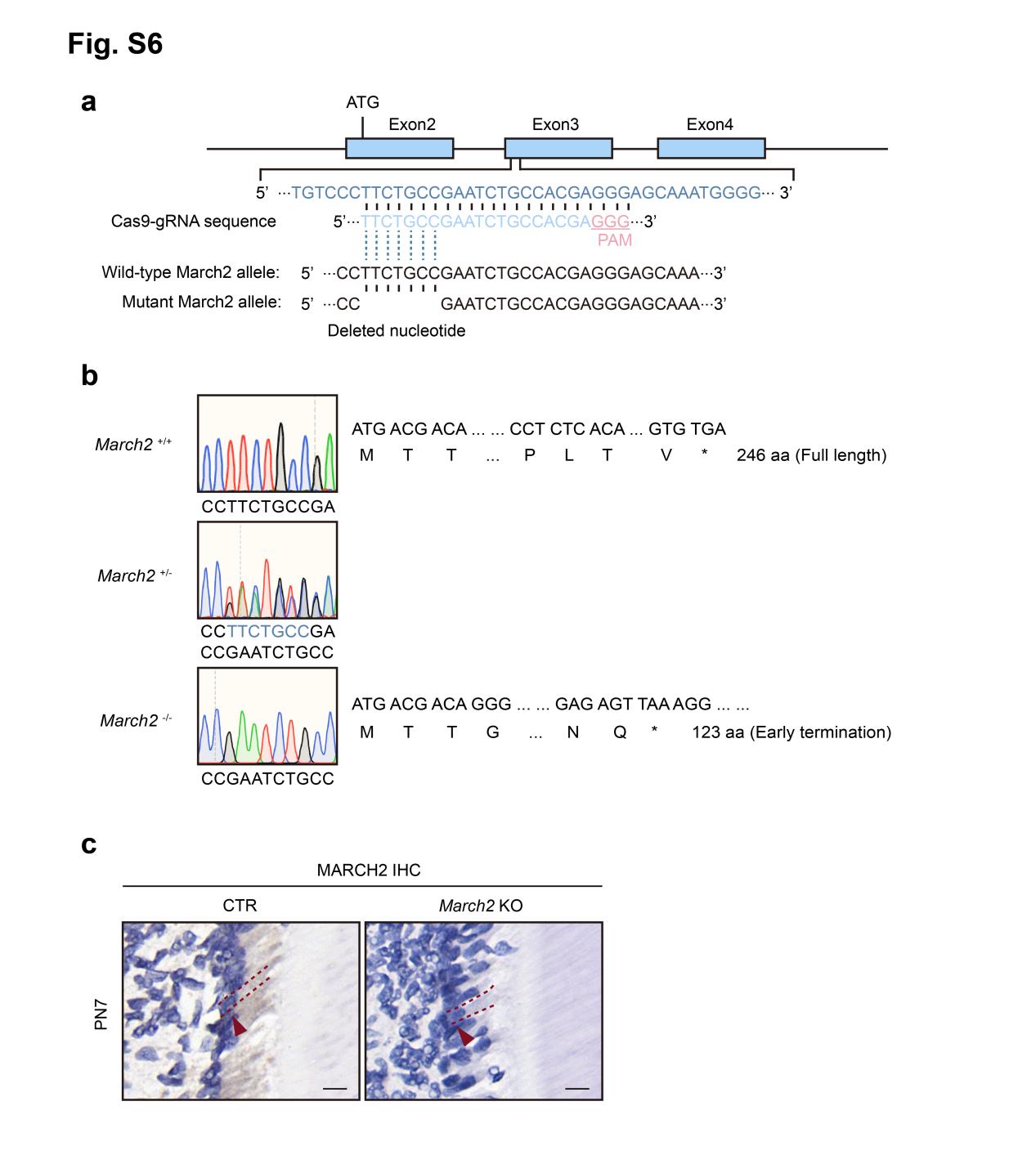


**Fig. S6** Construction of *March2* deficient mice and validation of the knockout efficiency. **a** A schematic diagram illustrating the targeting strategy used for generating *March2* deficient mice. **b** Sanger sequencing of the indicated genotypes revealed a 7 bp deletion in the genome of the *March2* deficient mice, resulting in a frameshift and premature termination of MARCH2 protein translation. **c** IHC shows that different from control littermate mice, *March2* deficient mice showed no MARCH2 expression in the odontoblast layer. Red arrows and red dashed lines indicate odontoblasts. CTR, control littermates; *March2* KO, *March2* deficient; Scale bar, 10 µm.


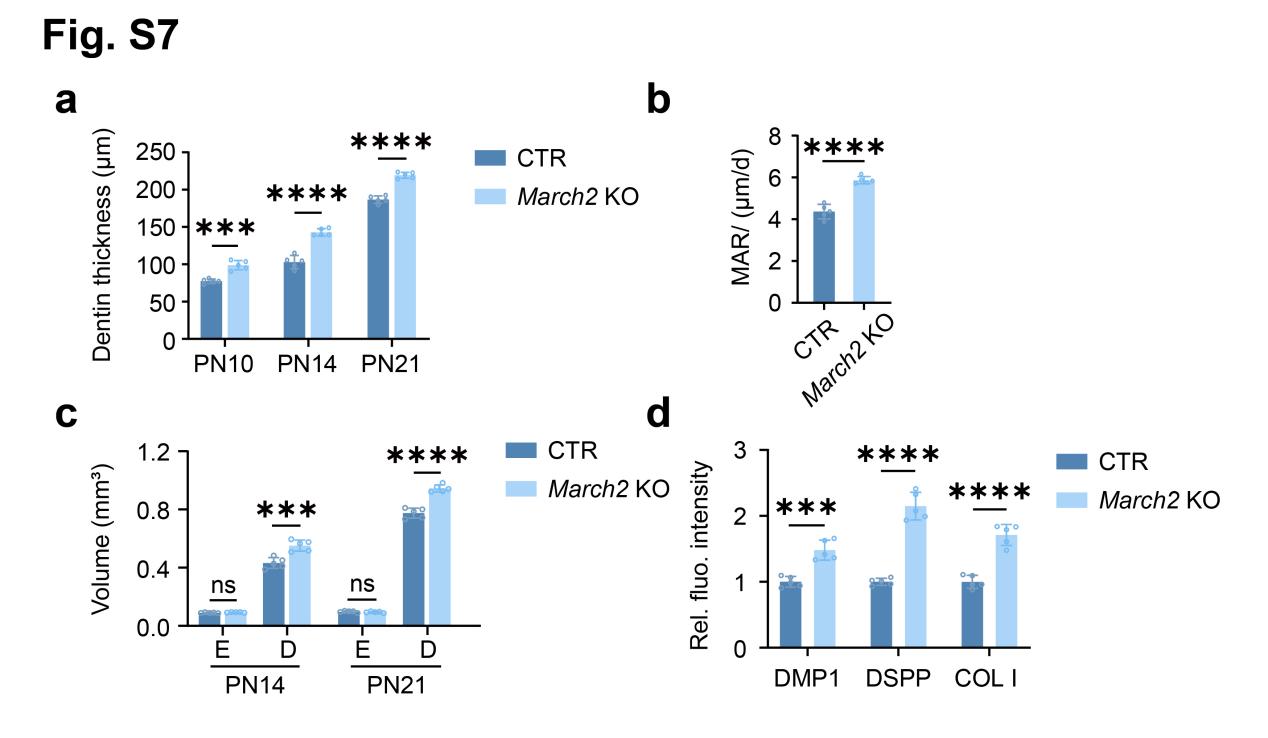


**Fig. S7** Supplementary quantification of dentin thicknesses, mineral apposition rates, enamel and dentin volumes and relative fluorescence intensity related to Fig. 3. **a** Quantification of the dentin thicknesses of molars in Fig. 3a. The dentin thickness was assessed in one out of every five consecutive sections from a total of five HE-stained samples. **b** Quantification of the mineral apposition rates of control littermates and *March2* deficient mice in Fig. 3b (n = 5). **c** Quantification of enamel and dentin volumes of the first mandibular molars from control littermates and *March2* deficient mice at PN14 and PN21 in Fig. 3d (n = 5). **d** Quantification of the relative fluorescence intensity of DMP1, DSPP and COL I in Fig. 3e (n = 5). CTR, control littermates; *March2* KO, *March2* deficient; E, enamel; D, dentin; MAR, mineral apposition rates. The statistical difference was analyzed by two-tailed unpaired Student’ s t-test (**a**, **b**, **c**, **d**), where ns denotes not significant; ****P* < 0.001; *****P* < 0.000 1.


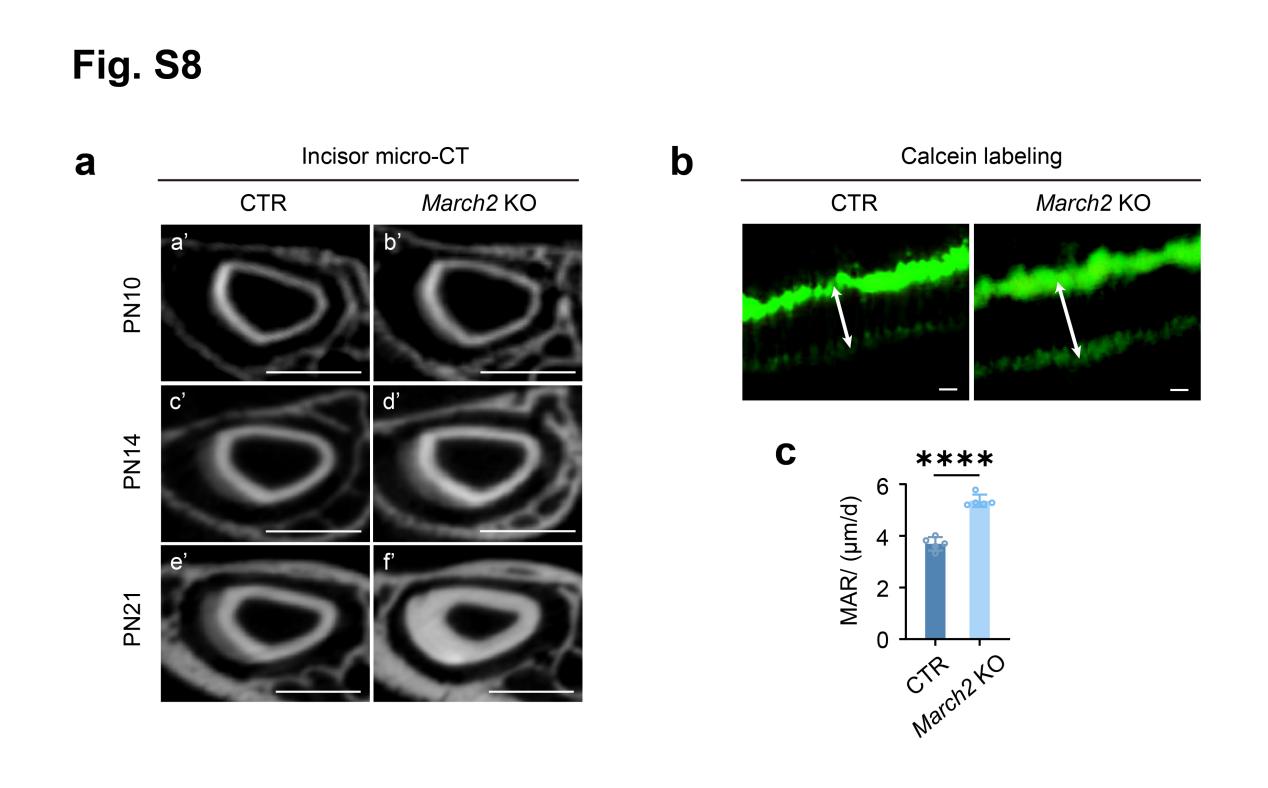


**Fig. S8** The incisors of *March2* deficient mice exhibited increased dentin thickness. **a** Micro-computed tomography (Micro-CT) images of the mandibular incisors in control littermates and *March2* deficient mice at PN10 (a’, b’), PN14 (c’, d’) and PN21 (e’, f’). Scale bar, 500 μm. **b** Calcein labeling was used to compare the dentin deposition rate in the mandibular incisors between control littermates and *March2* deficient mice. The white arrows indicate the dentin deposited in the period between drug administrations. Scale bar, 10 μm. **c** Quantification of the mineral apposition rates of dentin in the control littermates and *March2* deficient mice in (**b**) (n = 5). CTR, control littermates; *March2* KO, *March2* deficient; The statistical difference was analyzed by two-tailed unpaired Student’ s t-test (**c**), where *****P* < 0.000 1.

**
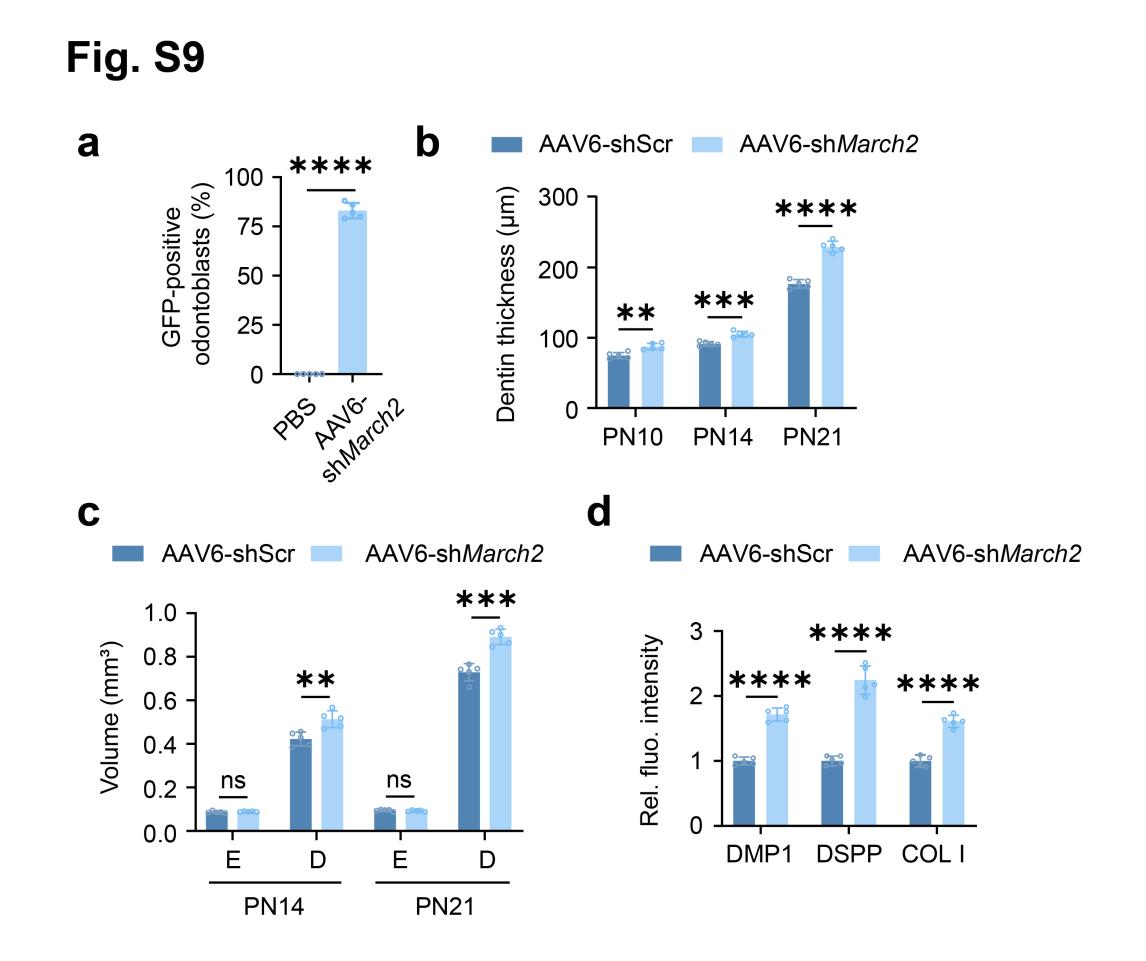
**

**Fig. S9** Supplementary quantification of the percentage of GFP-positive odontoblasts, dentin thicknesses, enamel and dentin volumes and relative fluorescence intensity related to Fig. 4. **a** Quantification of the percentage of GFP-positive odontoblasts shown in Fig. 4b (n = 5). **b** Quantification of the dentin thickness of molars in Fig. 4d. The dentin thickness was assessed in one out of every five consecutive sections from a total of five HE-stained samples. **c** Quantification of enamel and dentin volumes in Fig. 4f (n = 5). **d** Quantification of the relative fluorescence intensity of DMP1, DSPP and COL I in Fig. 4g (n = 5). E, enamel; D, dentin. The statistical difference was analyzed by two-tailed unpaired Student’ s t-test (**a**, **b**, **c**, **d**), where ns denotes not significant; ***P* < 0.01; ****P* < 0.001; *****P* < 0.000 1.


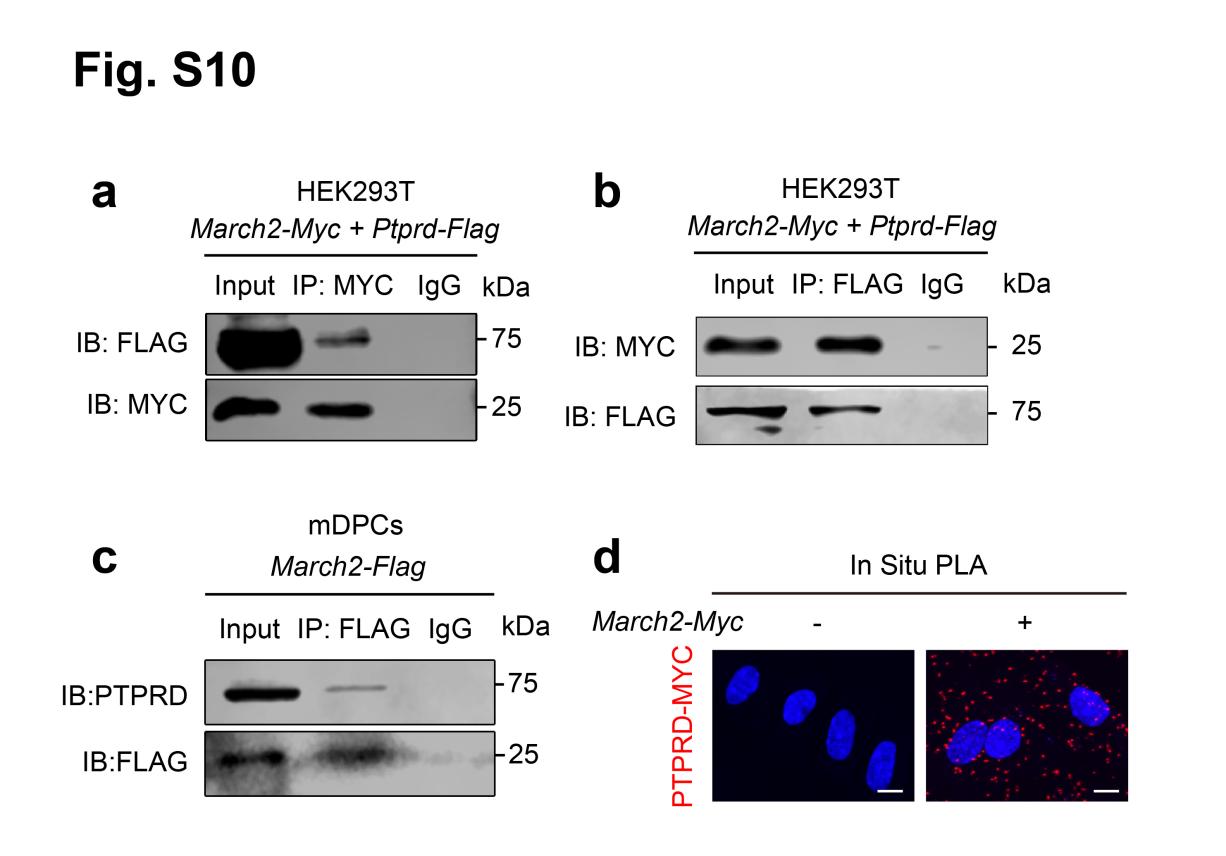


**Fig. S10** The physical interaction between MARCH2 and PTPRD **a**-**b** The interaction between overexpressed MARCH2 and PTPRD in human embryonic kidney 293T (HEK293T) cells detected by co-immunoprecipitation (co-IP) assays. **c** The interaction between overexpressed MARCH2 and endogenous PTPRD in cultured mDPCs detected by co-IP assays. **d** The physical proximity between overexpressed MARCH2 and endogenous PTPRD in cultured mDPCs was shown by in situ proximity ligation assay (PLA). Red punctate fluorescence indicates the physical proximity of two proteins.


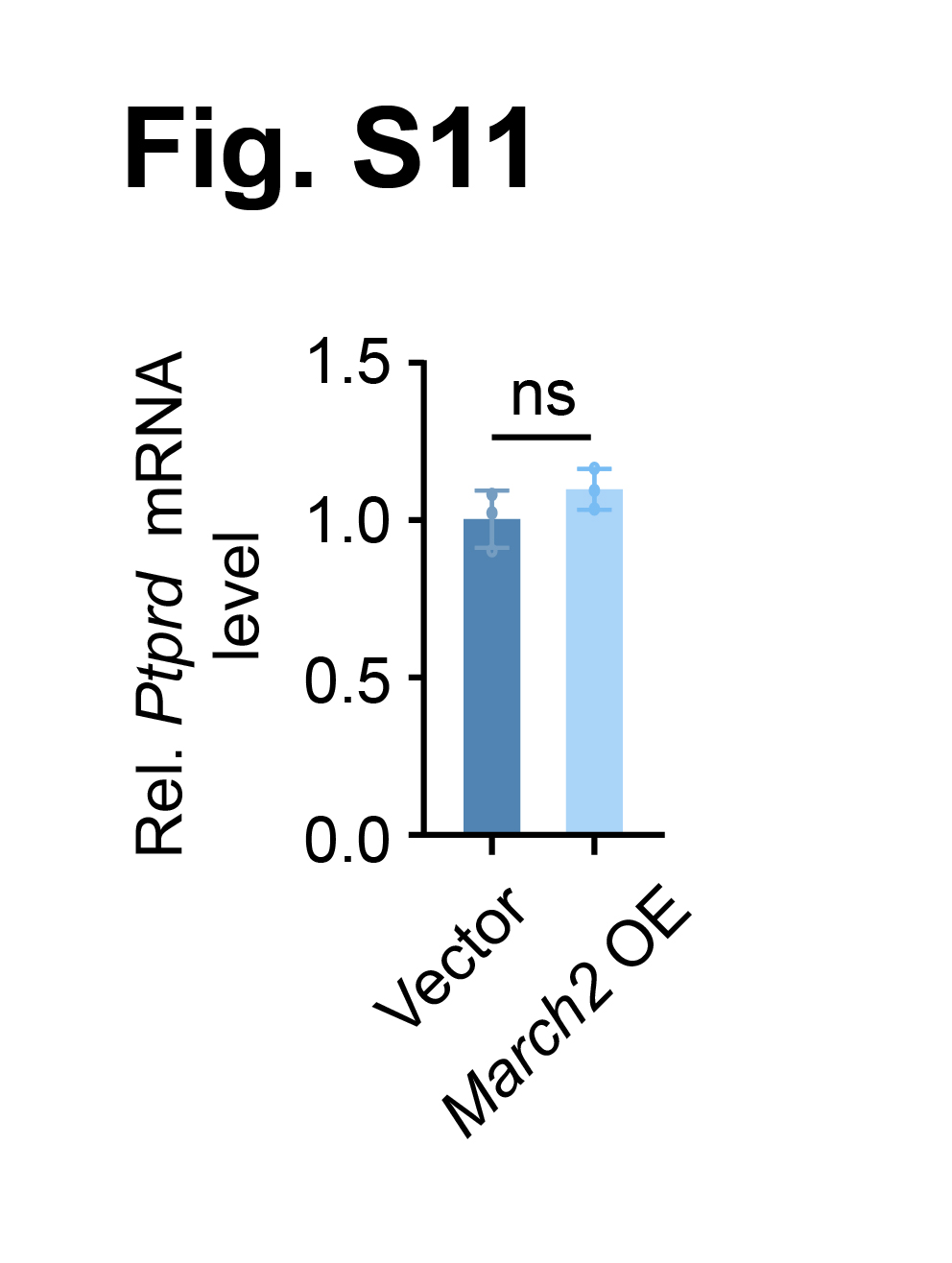


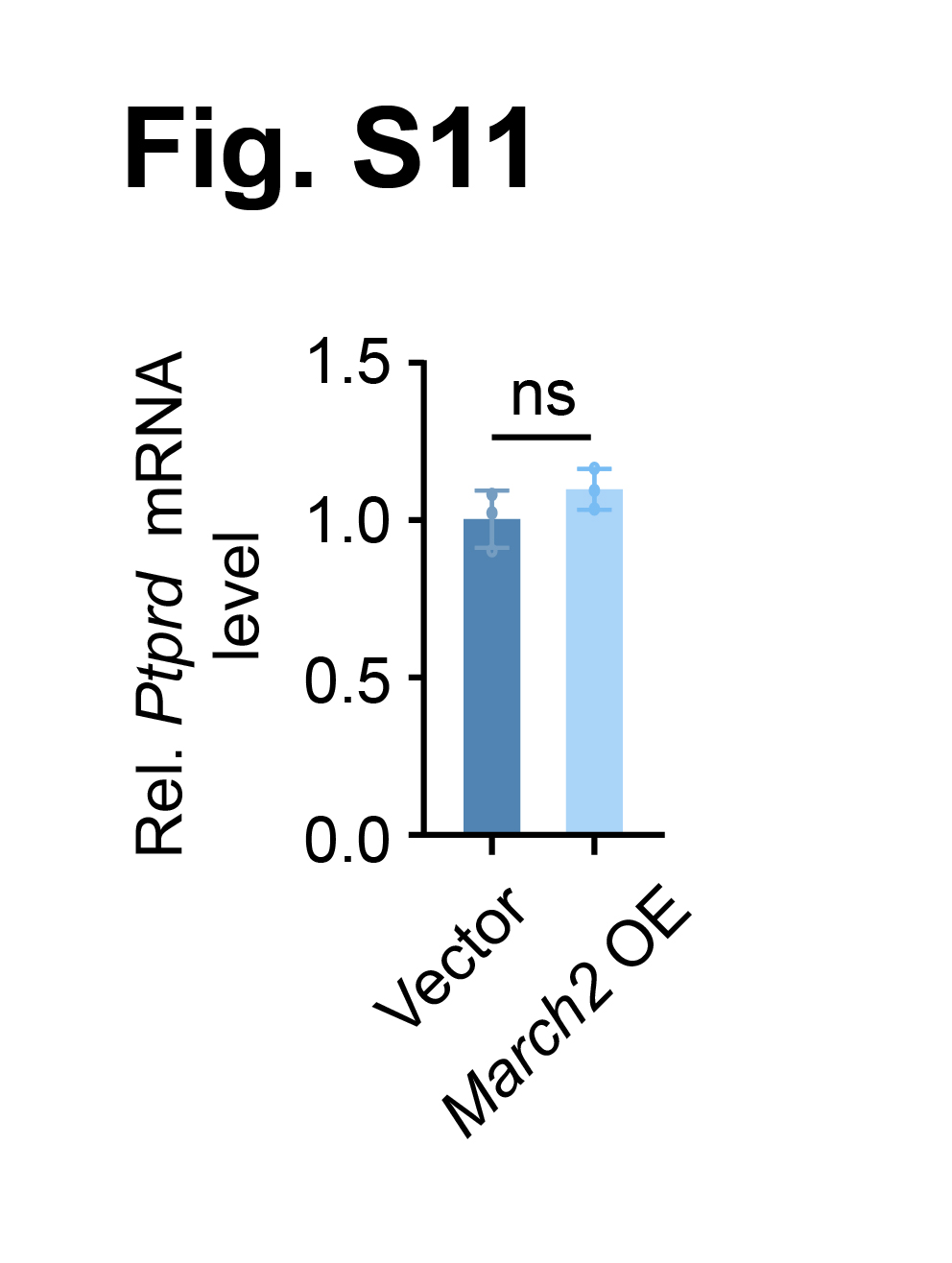


**Fig. S11** MARCH2 overexpression does not change *Ptprd* mRNA level in mDPCs (n = 3). The statistical difference was analyzed by two-tailed unpaired Student’ s t-test, where ns denotes not significant.

**
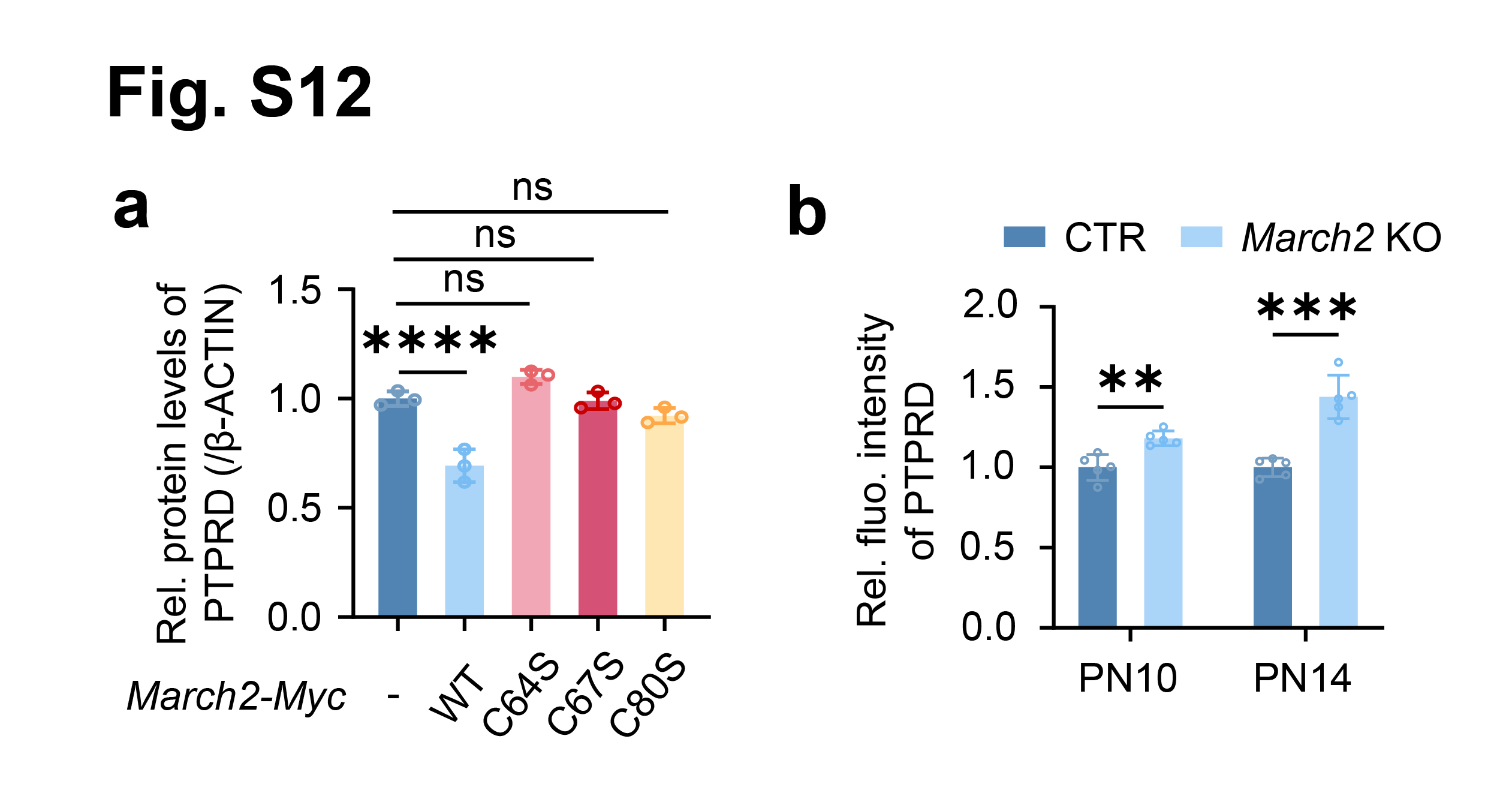
**

**
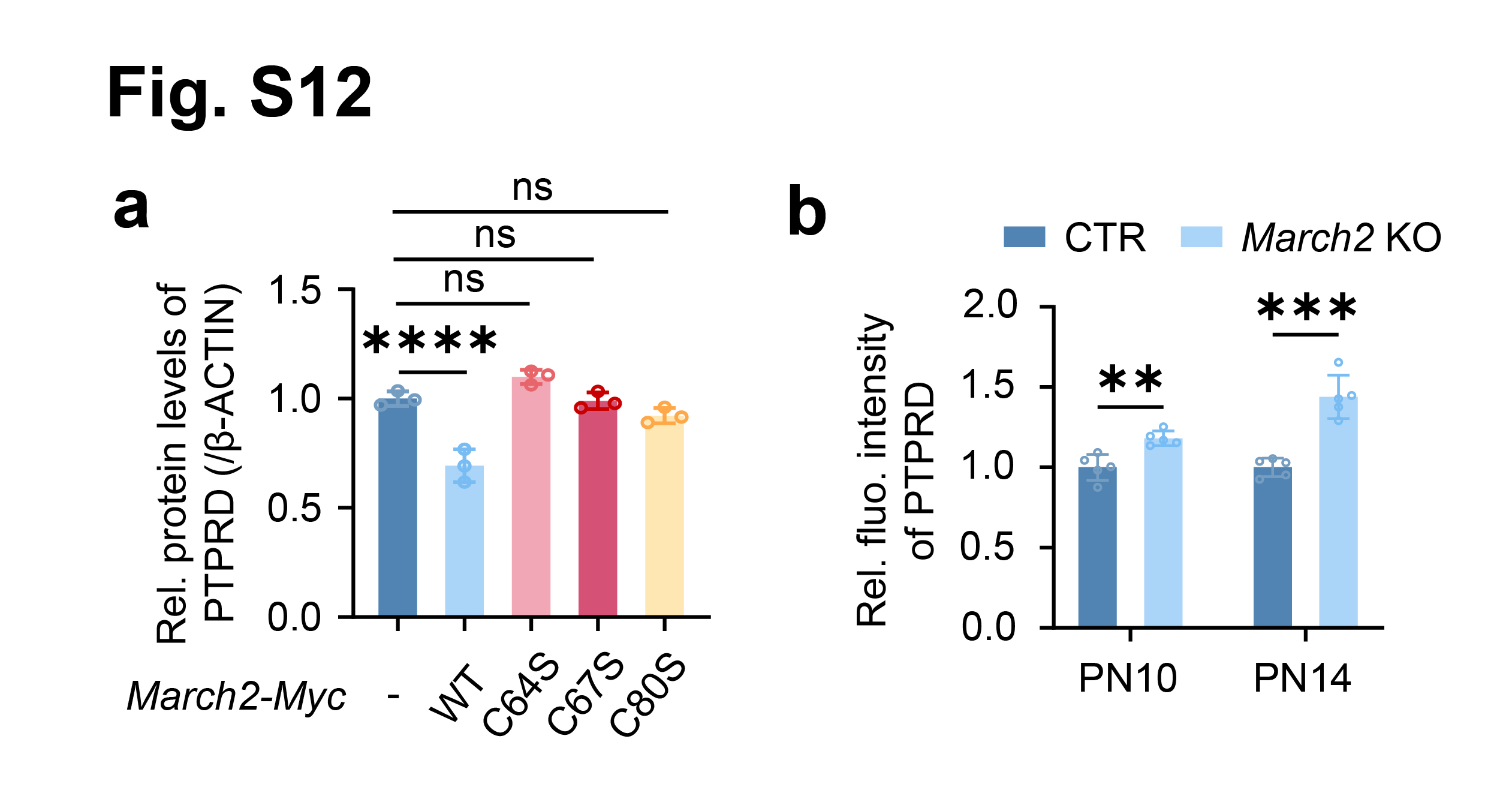
**

**Fig. S12** Supplementary quantification of WB analysis and IF staining related to Fig. 5. **a** Quantification of the relative protein levels of endogenous PTPRD in Fig. 5e (n = 3). **b**, Quantification of the relative fluorescence intensity of PTPRD in Fig. 5f (n = 5). The statistical difference was analyzed by two-tailed unpaired Student’ s t-test (**b**) and one-way ANOVA with Tukey’s post hoc test (**a**), where ns denotes not significant; ***P* < 0.01; ****P* < 0.001; *****P* < 0.000 1.

**
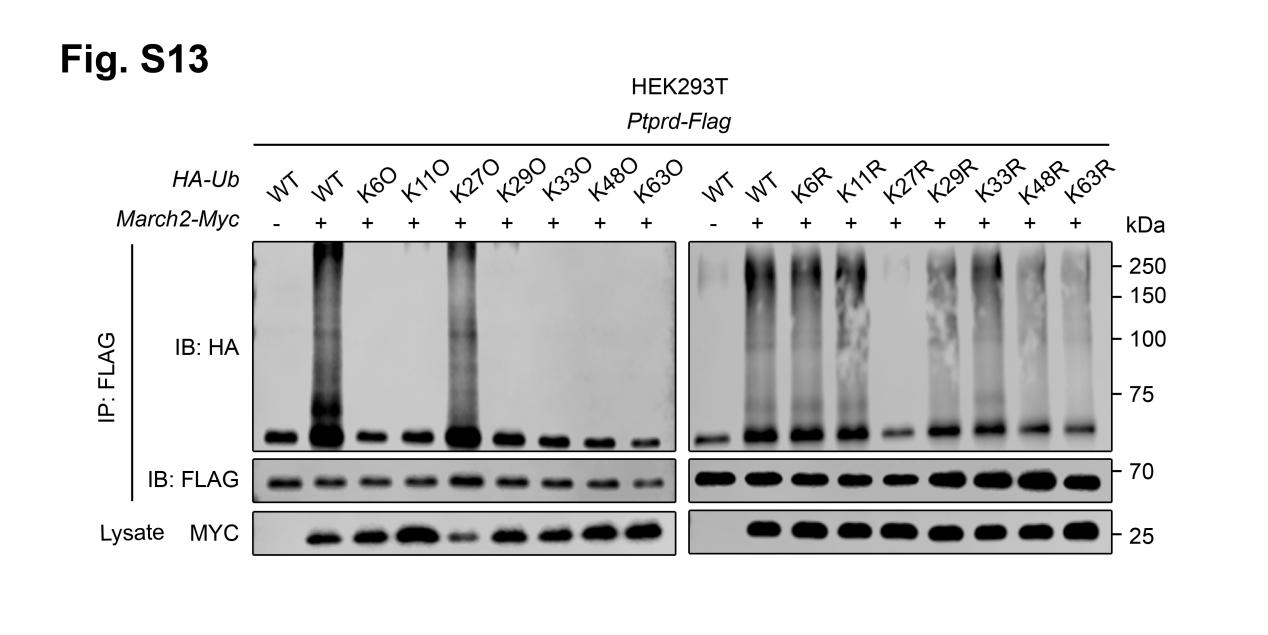
**

**Fig S13** MARCH2 specifically promoted K27-linked polyubiquitination of PTPRD. *Ubiquitin* or its mutant plasmids (KO or KR) were transfected into HEK293T cells together with *March2-Myc* and *Ptprd-Flag* plasmids. Co-IP assays show that MARCH2 mediates the K27-polyubiquitination of PTPRD. KO, containing only a single lysine residue; KR, only one lysine residue was mutated to arginine.

**
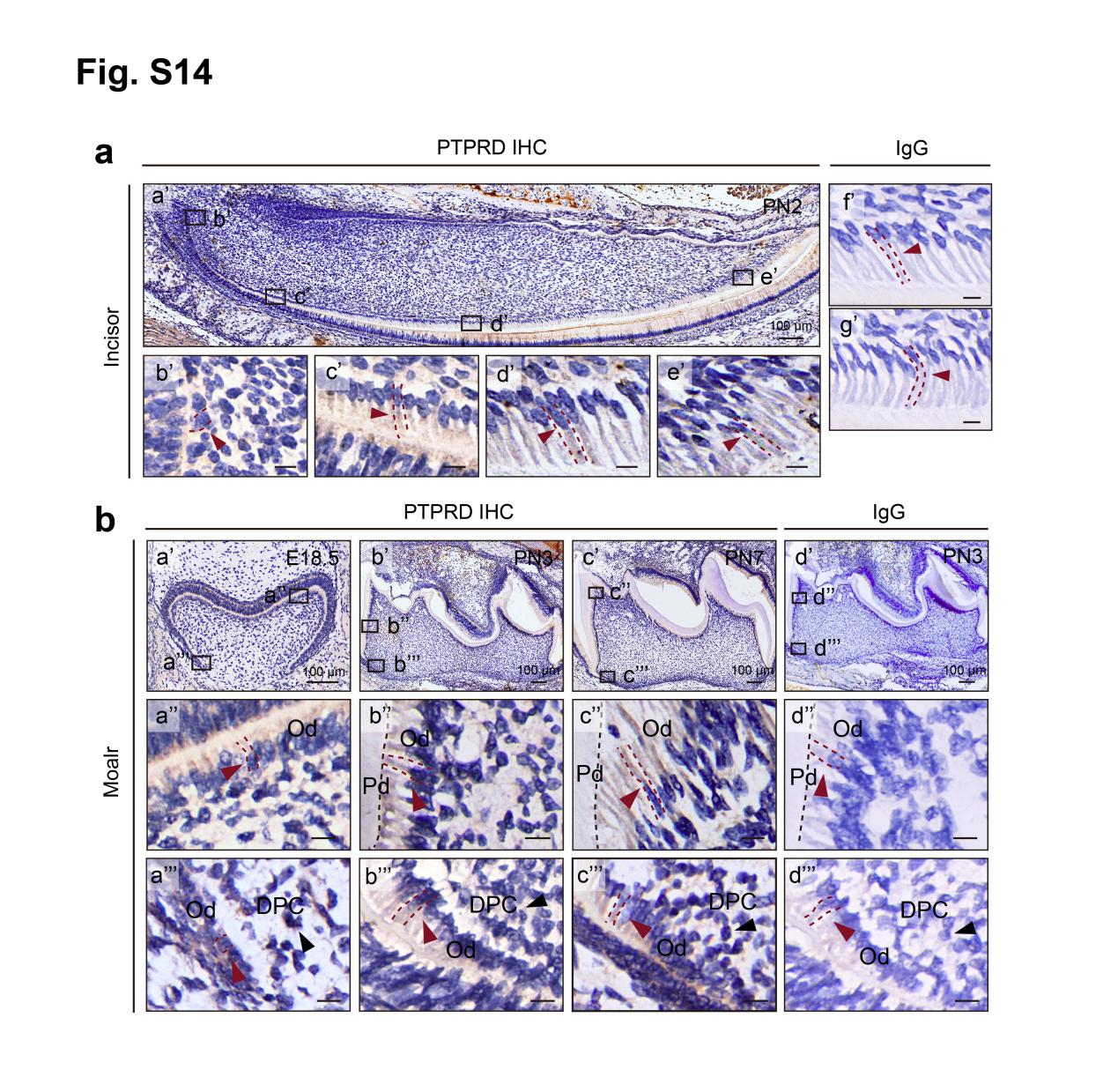
Fig. S14** The expression pattern of PTPRD during odontoblast differentiation *in vivo*. **a** IHC of PTPRD in mouse incisors at PN2. b’, c’, d’, and e’ are enlarged views of the rectangles in a’. Red arrows and red dashed lines indicate dental papilla cell (b’) and odontoblasts (c’-g’). f’ and g’ represent negative controls using nonimmune IgG instead of the primary antibody. **b** IHC of PTPRD in mouse molars from E18.5 to PN7. d’ represents negative control using nonimmune IgG instead of the primary antibody. a’’-d’’ and a’’’-d’’’ are enlarged views of the rectangles in a’-d’. Red arrows and red dashed lines mark the odontoblasts. Black arrows mark the dental papilla cells. The black dotted lines represent the boundaries between pre-dentin and odontoblast layer. Pd, pre-dentin; Od, odontoblasts; DPC, dental papilla cell. Except for the labeled scale bars, all others are 10 µm.

**
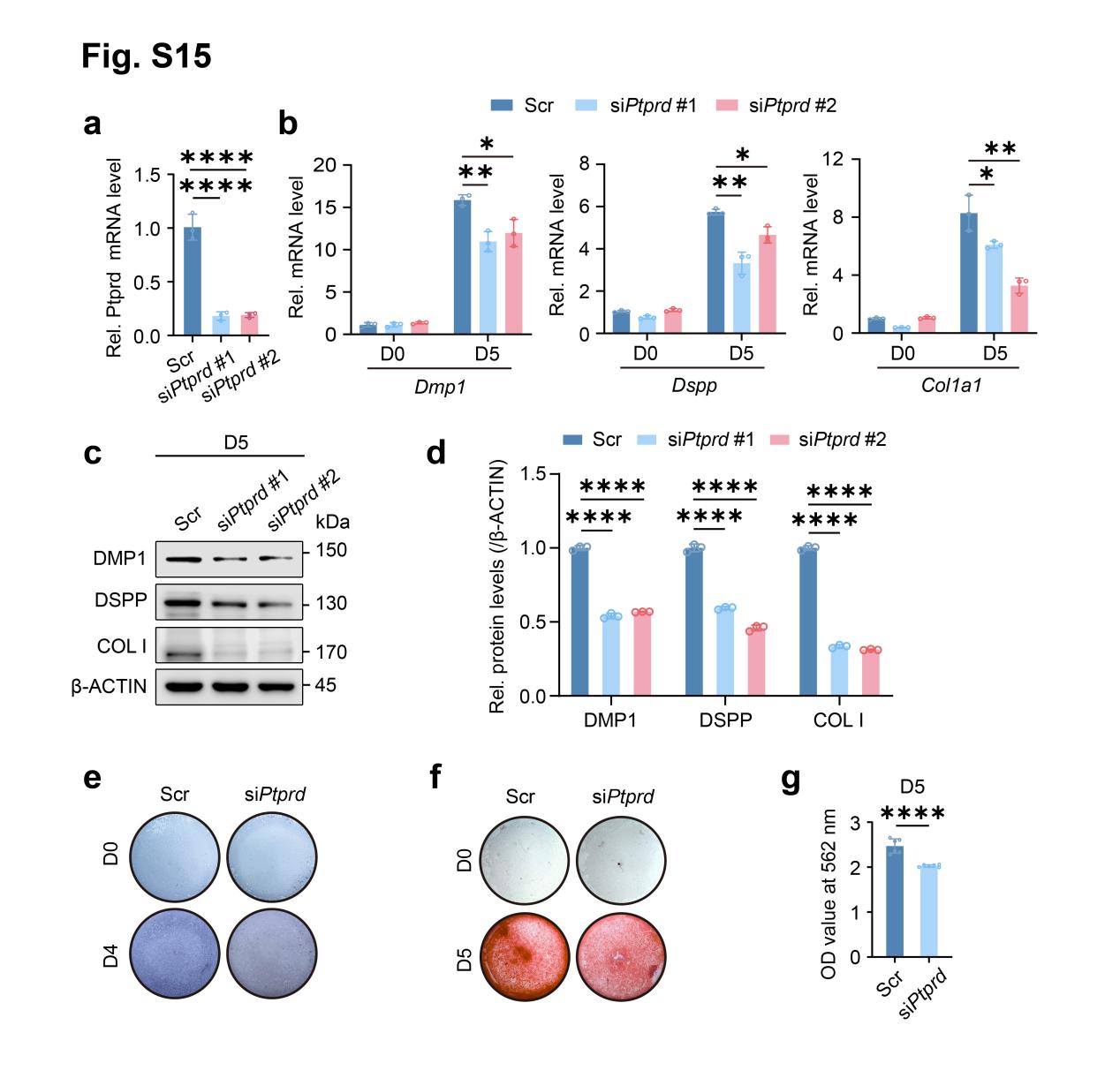
Fig. S15** PTPRD promotes the odontoblastic differentiation of mDPCs *in vitro*. **a** The knockdown efficiency of *Ptprd* siRNAs in mDPCs assessed by RT-qPCR. **b** Effects of *Ptprd* knockdown on mRNA expression of *Dmp1*, *Dspp* and *Col1a1* after differentiation induction for 5 d in mDPCs. **c** Effects of *Ptprd* knockdown on protein levels of DMP1, DSPP and COL I after differentiation induction for 5 d in mDPCs. **d** Quantification of the relative protein expression levels of DMP1, DSPP and COL I in (**c**) (n = 3). **e** The effects of *Ptprd* knockdown on the ALP activity detected by ALP staining. **f** The effects of *Ptprd* knockdown on the mineralized nodule formation measured by ARS staining in cultured mDPCs. **g** Semi-quantitative analysis of the OD value of the ARS-stained cells in (**f**) (n = 6). D, differentiation induction. The statistical difference was analyzed by two-tailed unpaired Student’ s t-test (**g**) and one-way ANOVA with Tukey’s post hoc test (**a**, **b**, **d**), where **P* < 0.05; ***P* < 0.01; *****P* < 0.000 1.

**
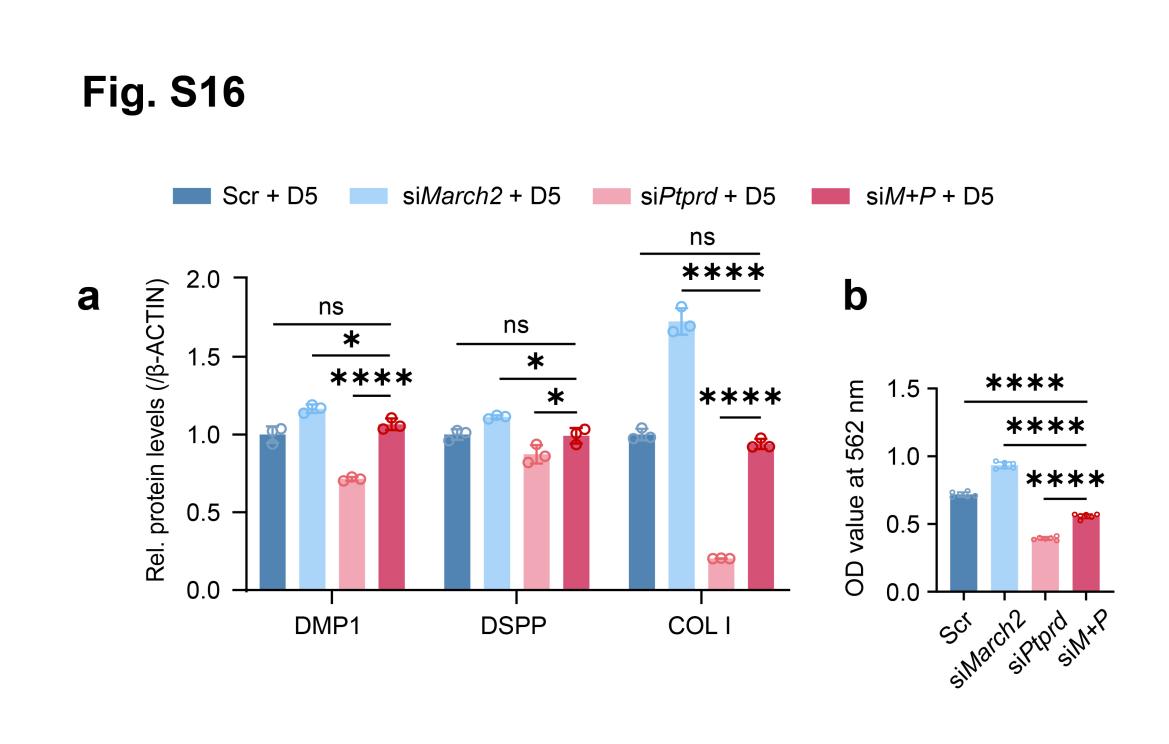
**

**Fig. S16** Supplementary quantification of WB analysis and ARS staining related to Fig. 7. **a** Quantification of the relative protein expression levels of DMP1, DSPP and COL I in Fig 7b (n = 3). **b** Semi-quantitative assessment of the OD values of cells stained with ARS in Fig 7d (n = 6). D, differentiation induction. The statistical difference was analyzed by one-way ANOVA with Dunnett’s post hoc test (**a**, **b**), where ns denotes not significant; **P* < 0.05; *****P* < 0.000 1.

**REFERENCES**

1 Jing, J. J. *et al.* Spatiotemporal single-cell regulatory atlas reveals neural crest lineage diversification and cellular function during tooth morphogenesis. *Nat. Commun.* **13**, 14 (2022). <https://doi.org:10.1038/s41467-022-32490-y>

2 Zheng, H., Fu, J., Chen, Z., Yang, G. & Yuan, G. Dlx3 Ubiquitination by Nuclear Mdm2 Is Essential for Dentinogenesis in Mice. *J. Dent. Res.* **101**, 1064-1074 (2022). <https://doi.org:10.1177/00220345221077202>
