# Supplementary material for: MARCH2 suppresses odontoblast differentiation by polyubiquitinating PTPRD
Source: Int J Oral Sci. 2026 Jan 10;18:5. doi: 10.1038/s41368-025-00407-2 (PMC12789588; doi:10.1038/s41368-025-00407-2)
Supplement: Supplementary file 2 — Raw western blot data [file 41368_2025_407_MOESM2_ESM.pdf]

Fig. 2c

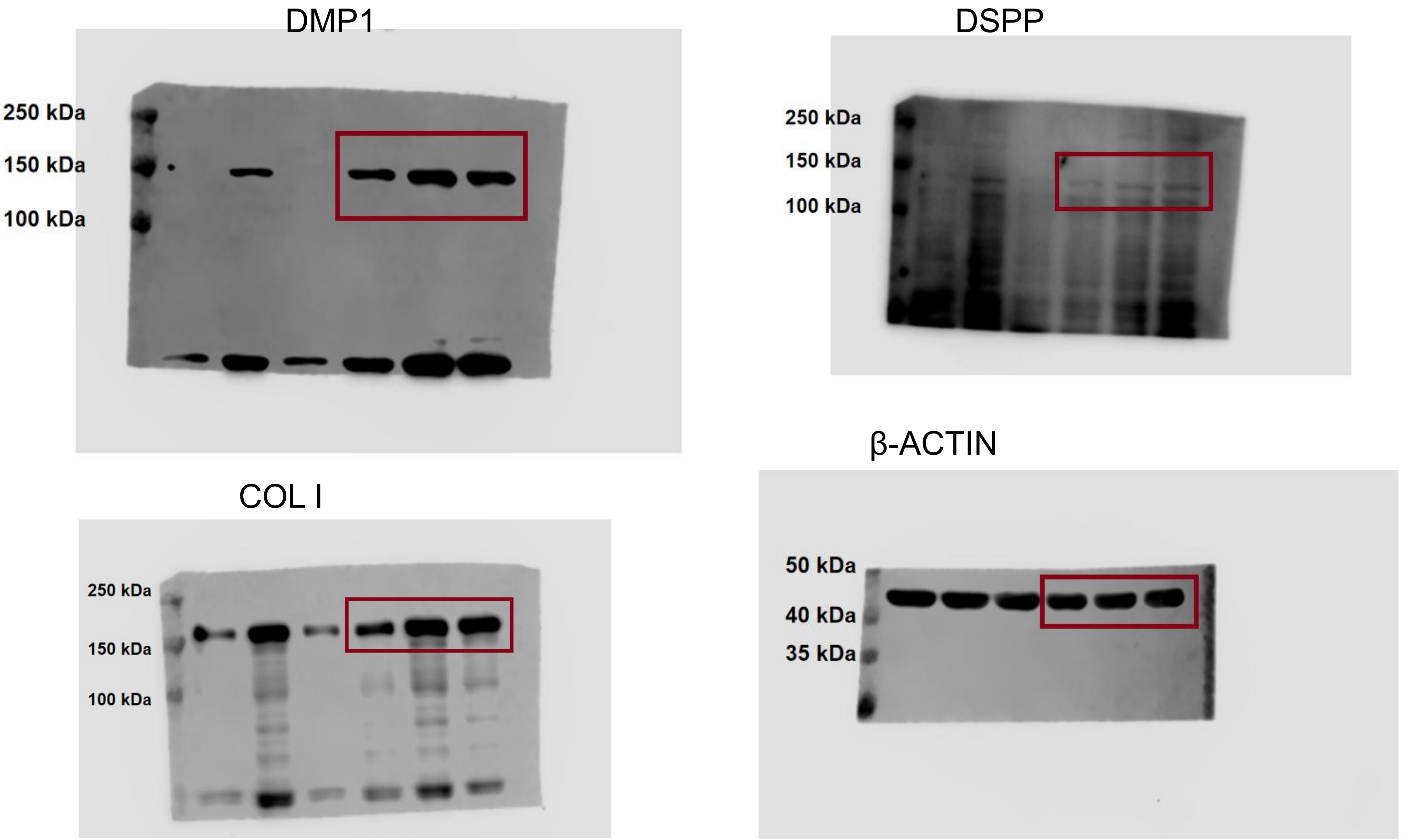
DMP1
250 kDa
150 kDa
100 kDa
DSPP
250 kDa
150 kDa
100 kDa
β-ACTIN
50 kDa
40 kDa
35 kDa
COL I
250 kDa
150 kDa
100 kDa

Fig. 5a

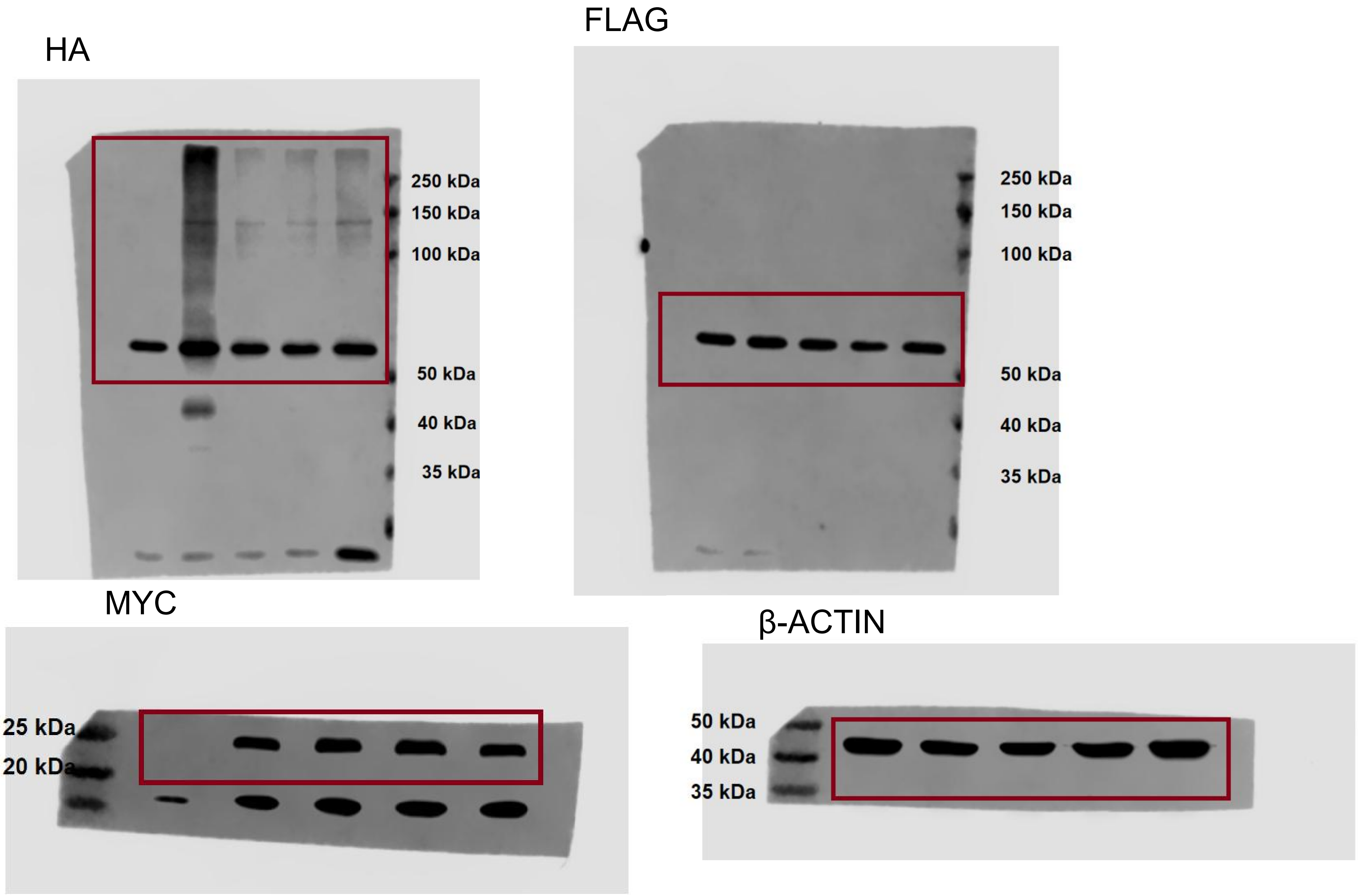

HA
250 kDa
150 kDa
100 kDa
50 kDa
40 kDa
35 kDa
FLAG
250 kDa
150 kDa
100 kDa
50 kDa
40 kDa
35 kDa
MYC
25 kDa
20 kDa
β-ACTIN
50 kDa
40 kDa
35 kDa

Fig. 5c

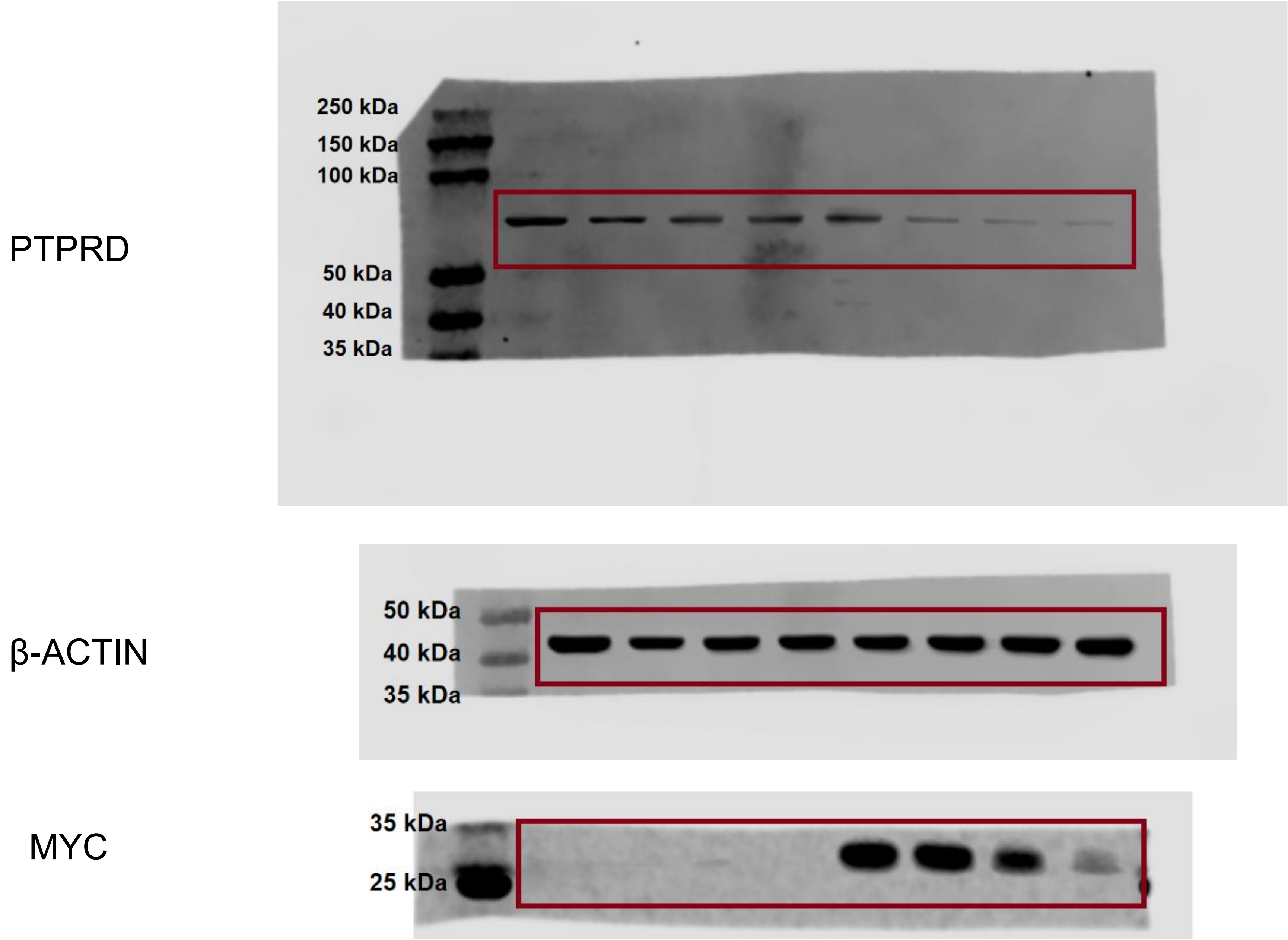
PTPRD
250 kDa
150 kDa
100 kDa
50 kDa
40 kDa
35 kDa
β-ACTIN
50 kDa
40 kDa
35 kDa
MYC
35 kDa
25 kDa

Fig. 5e

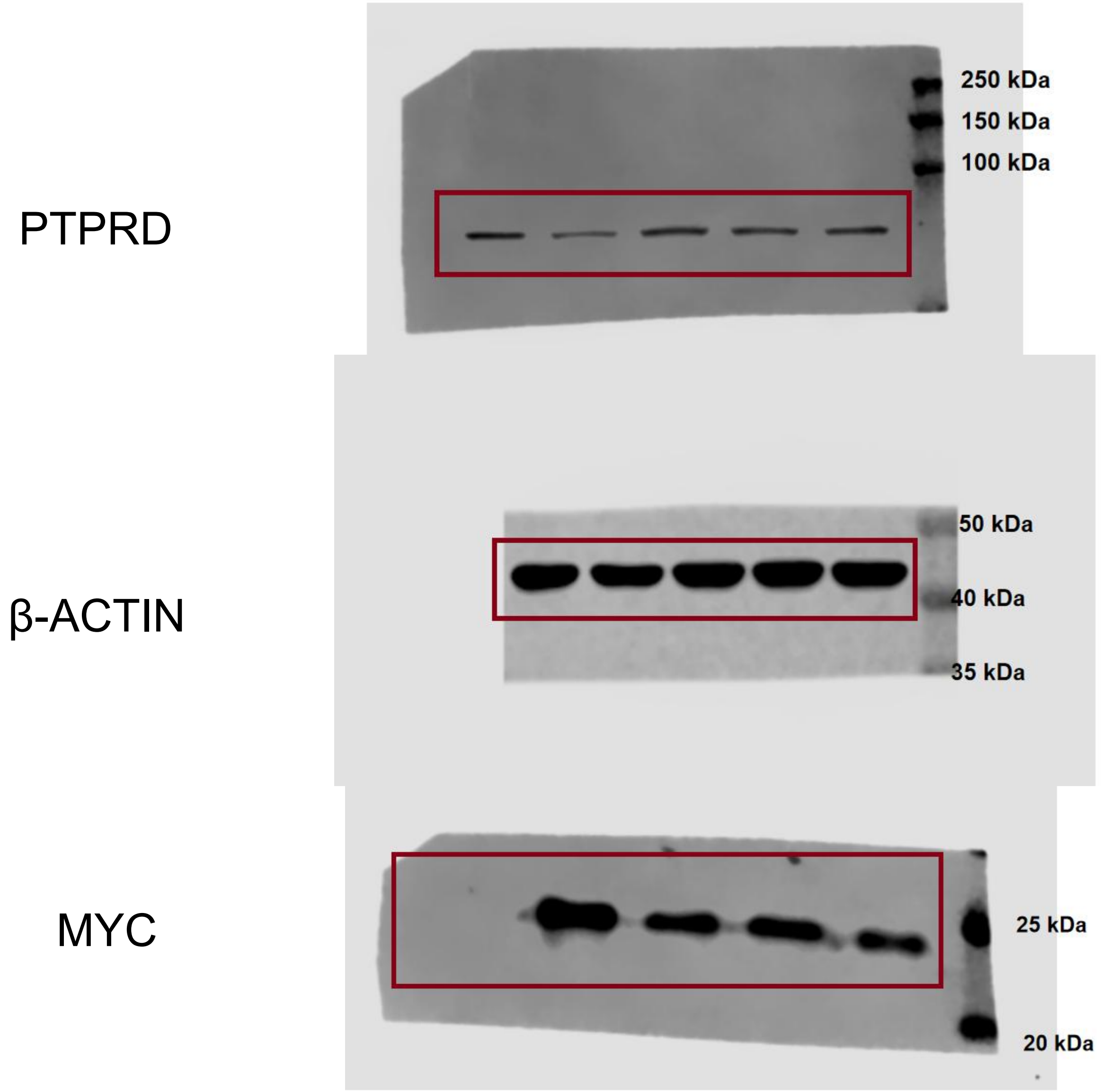
PTPRD
250 kDa
150 kDa
100 kDa
β-ACTIN
50 kDa
40 kDa
35 kDa
MYC
25 kDa
20 kDa

Fig. 6a

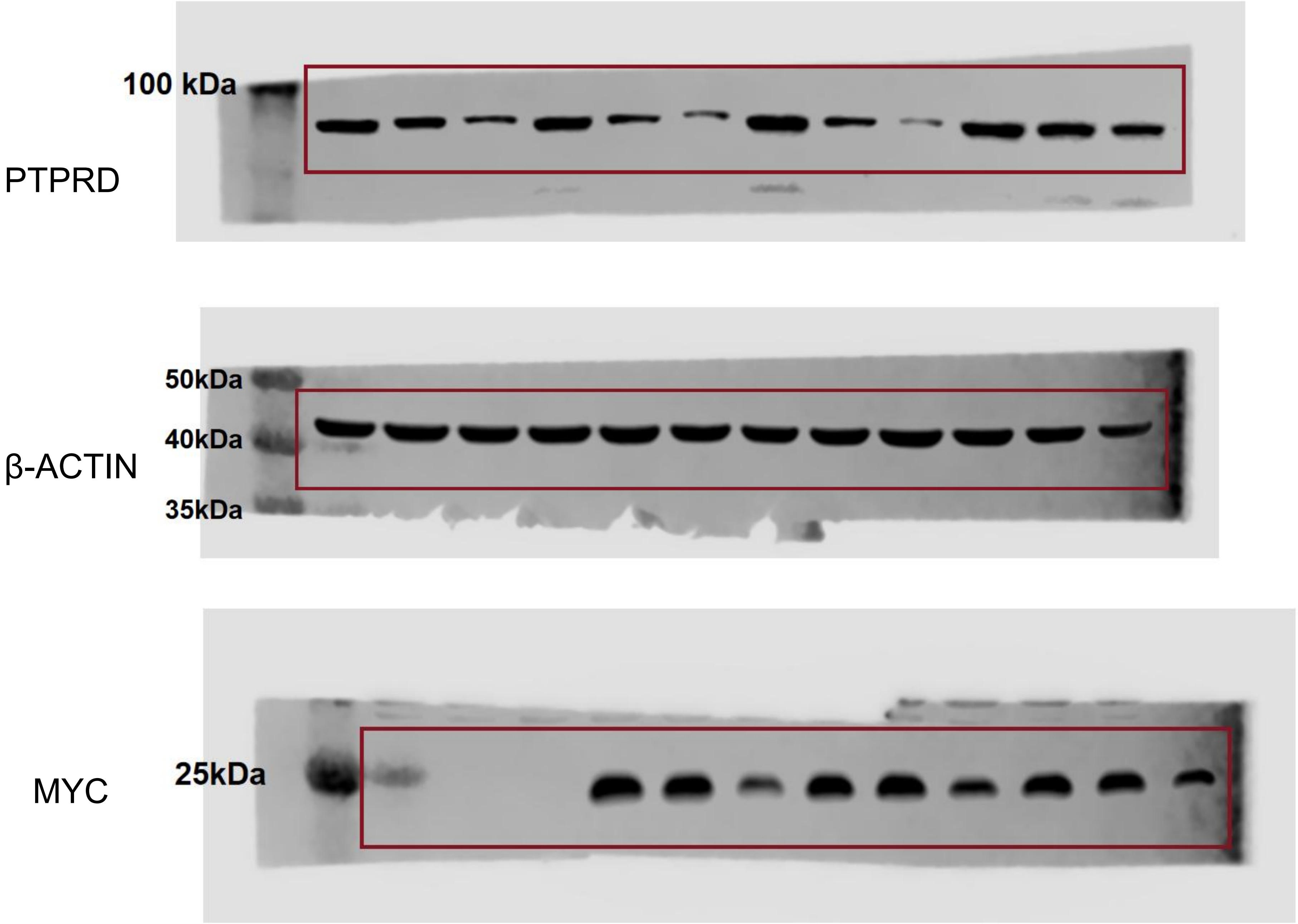

Fig. 7b

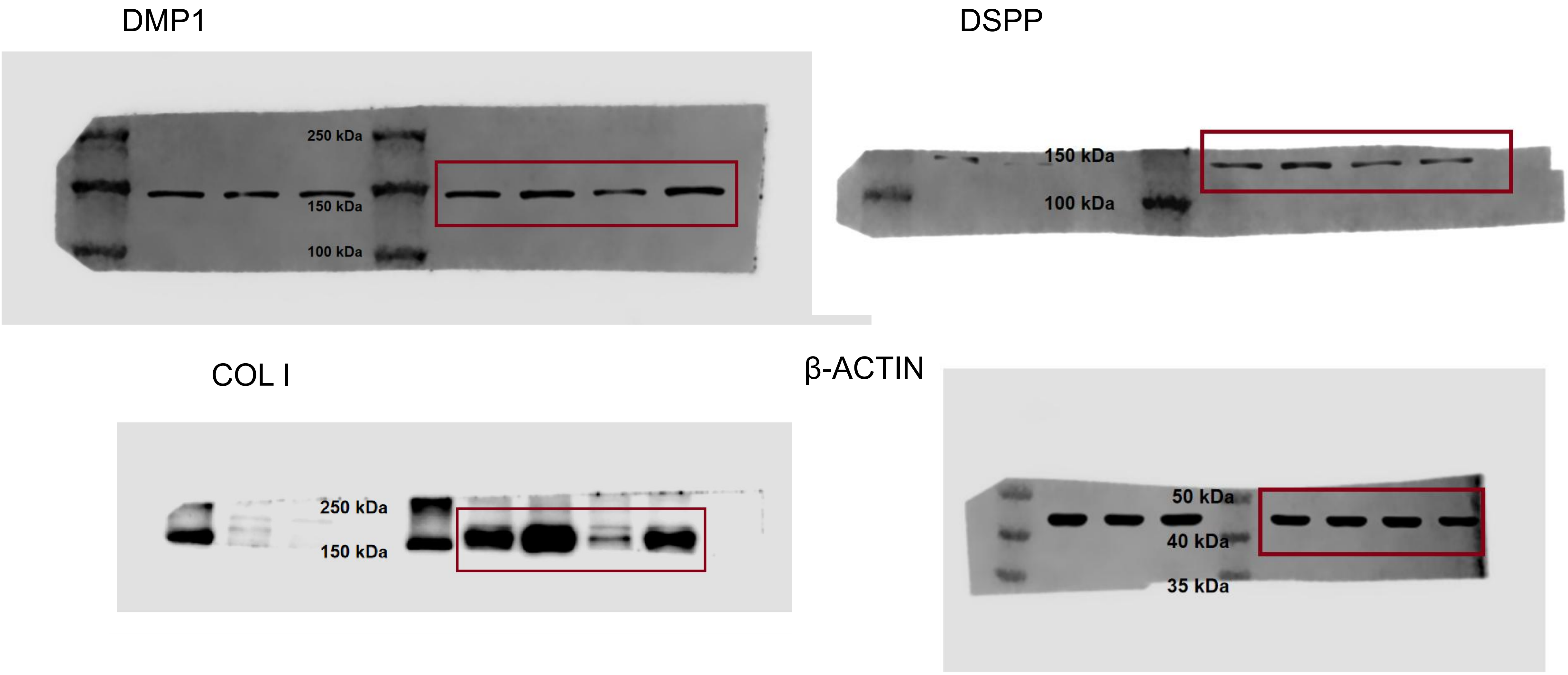

DMP1
250 kDa
150 kDa
100 kDa
DSPP
150 kDa
100 kDa
COL I
250 kDa
150 kDa
β-ACTIN
50 kDa
40 kDa
35 kDa

Fig. S1c

MARCH2

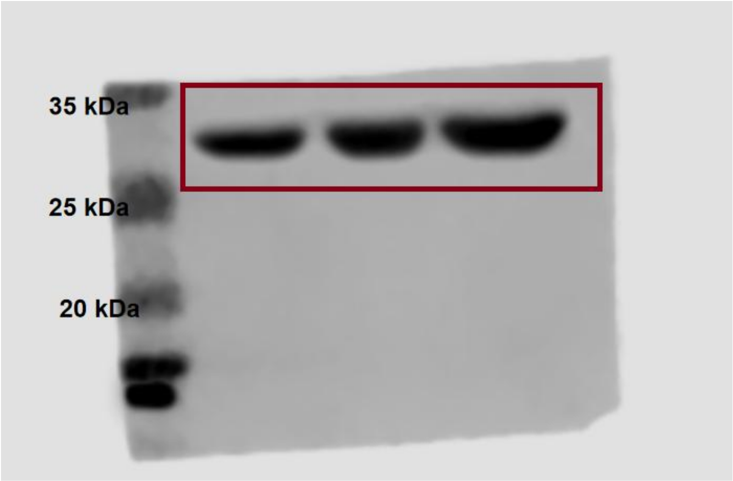

β-ACTIN

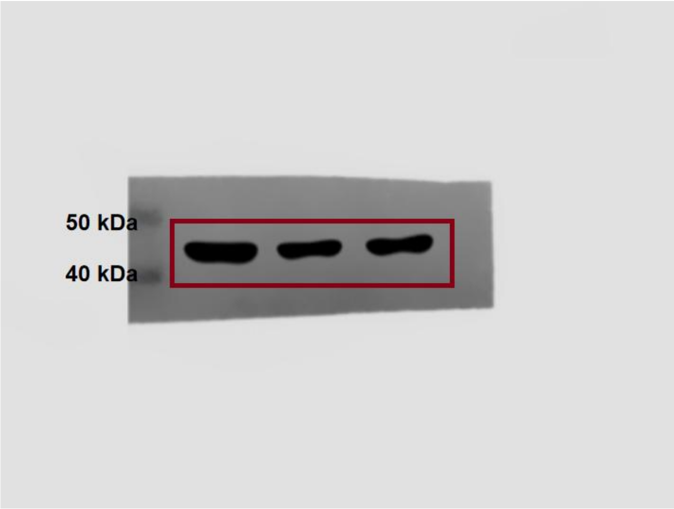

Fig. S5a

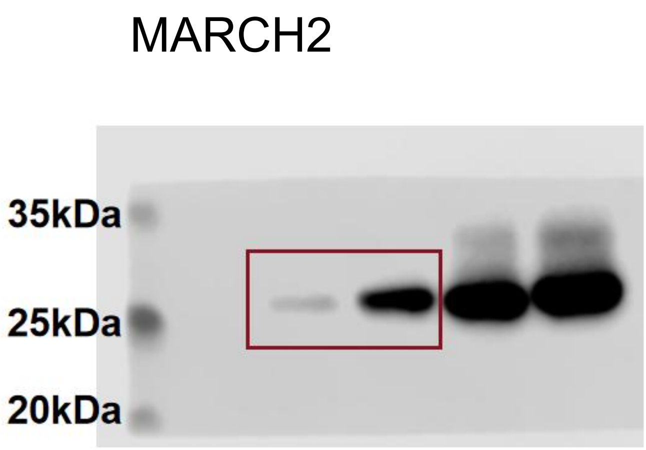

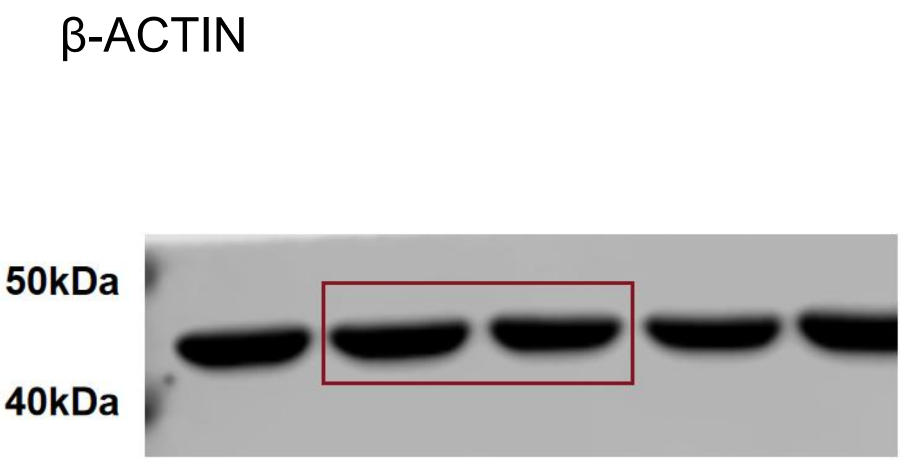

Fig. S5c

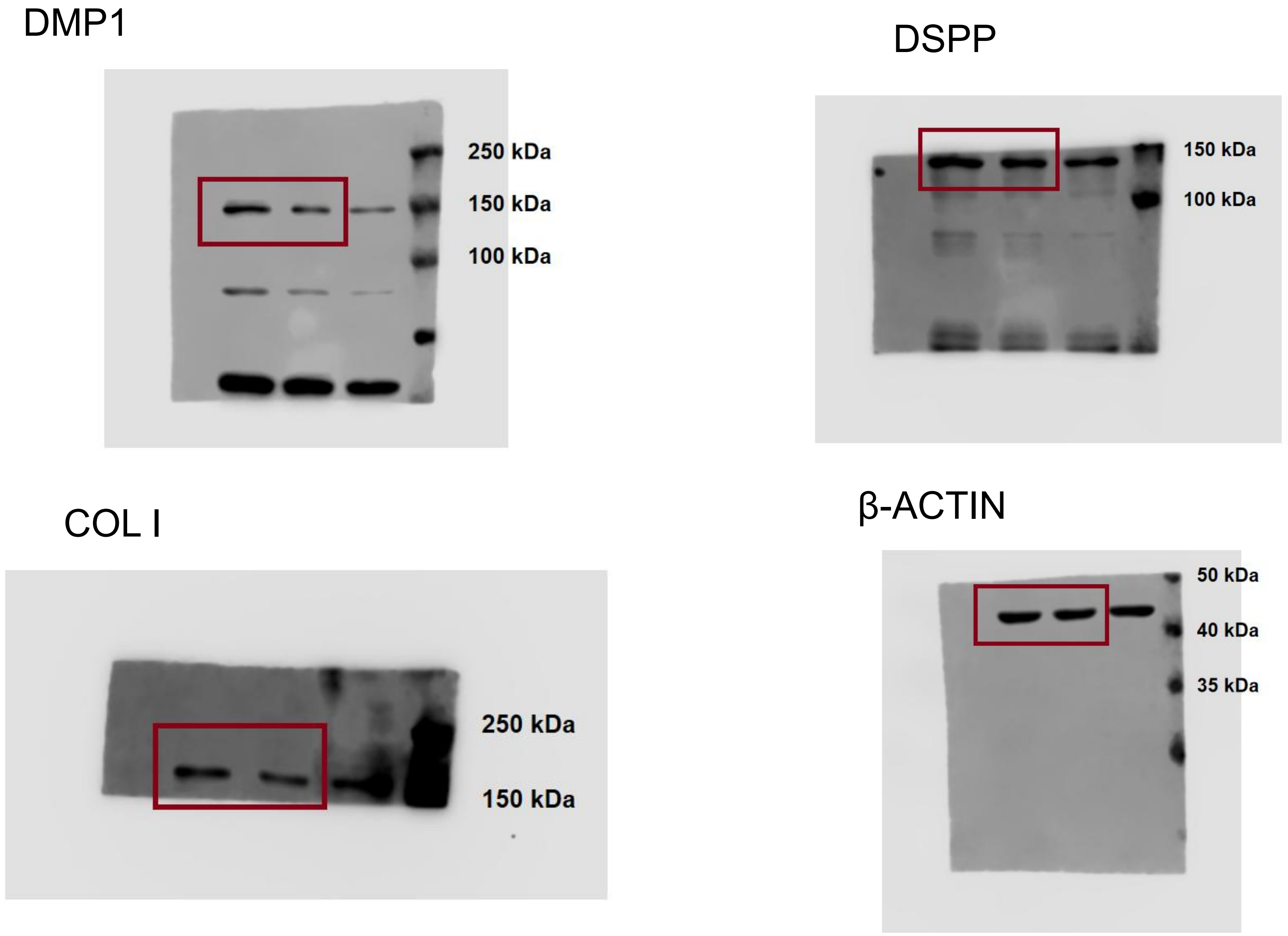
DMP1
250 kDa
150 kDa
100 kDa
DSPP
150 kDa
100 kDa
COL I
250 kDa
150 kDa
β-ACTIN
50 kDa
40 kDa
35 kDa

Fig. S10a

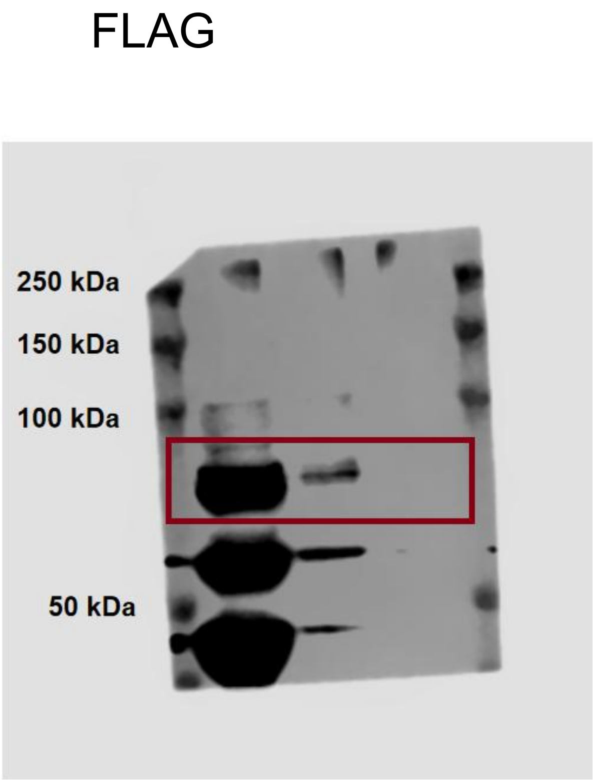

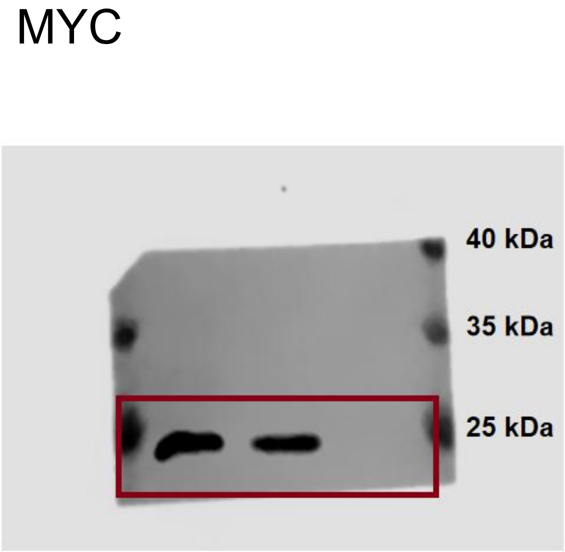

Fig. S10b

FLAG

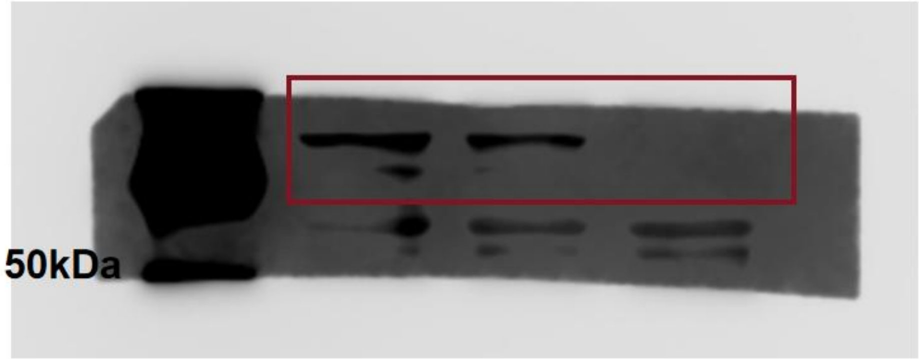

MYC

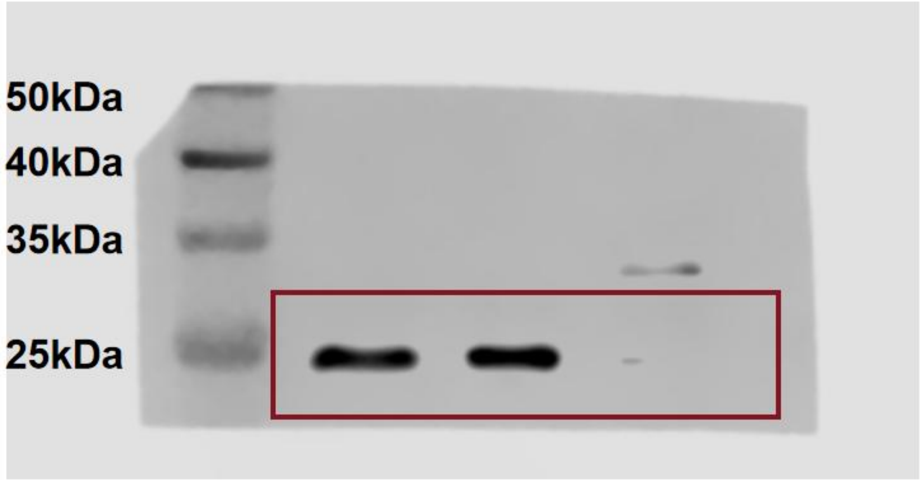

Fig. S10c

PTPRD

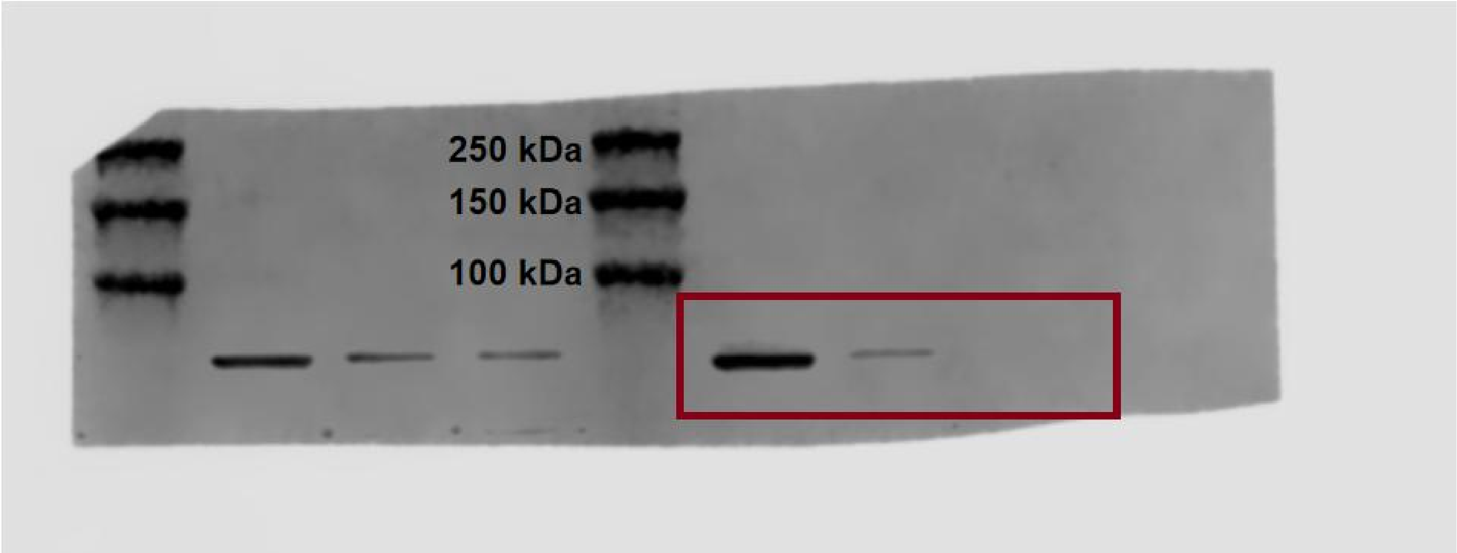

FLAG

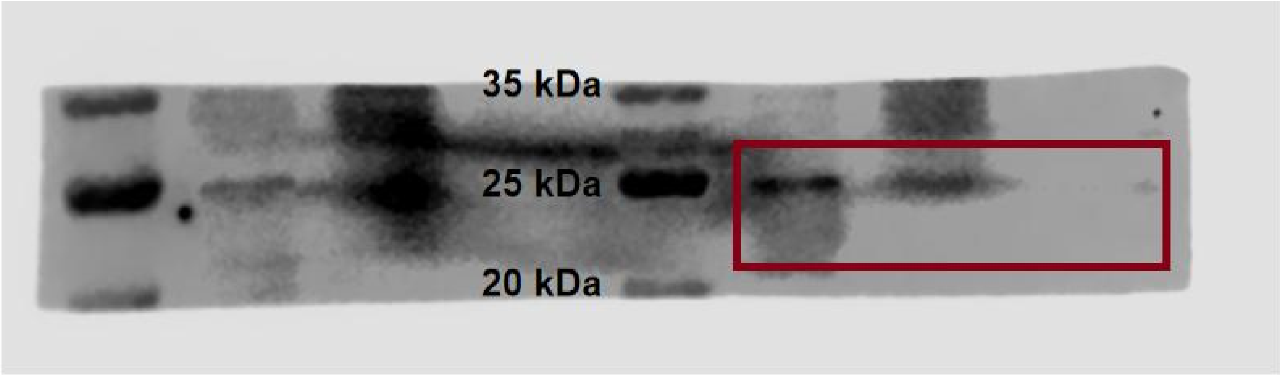

Fig. 13

KO

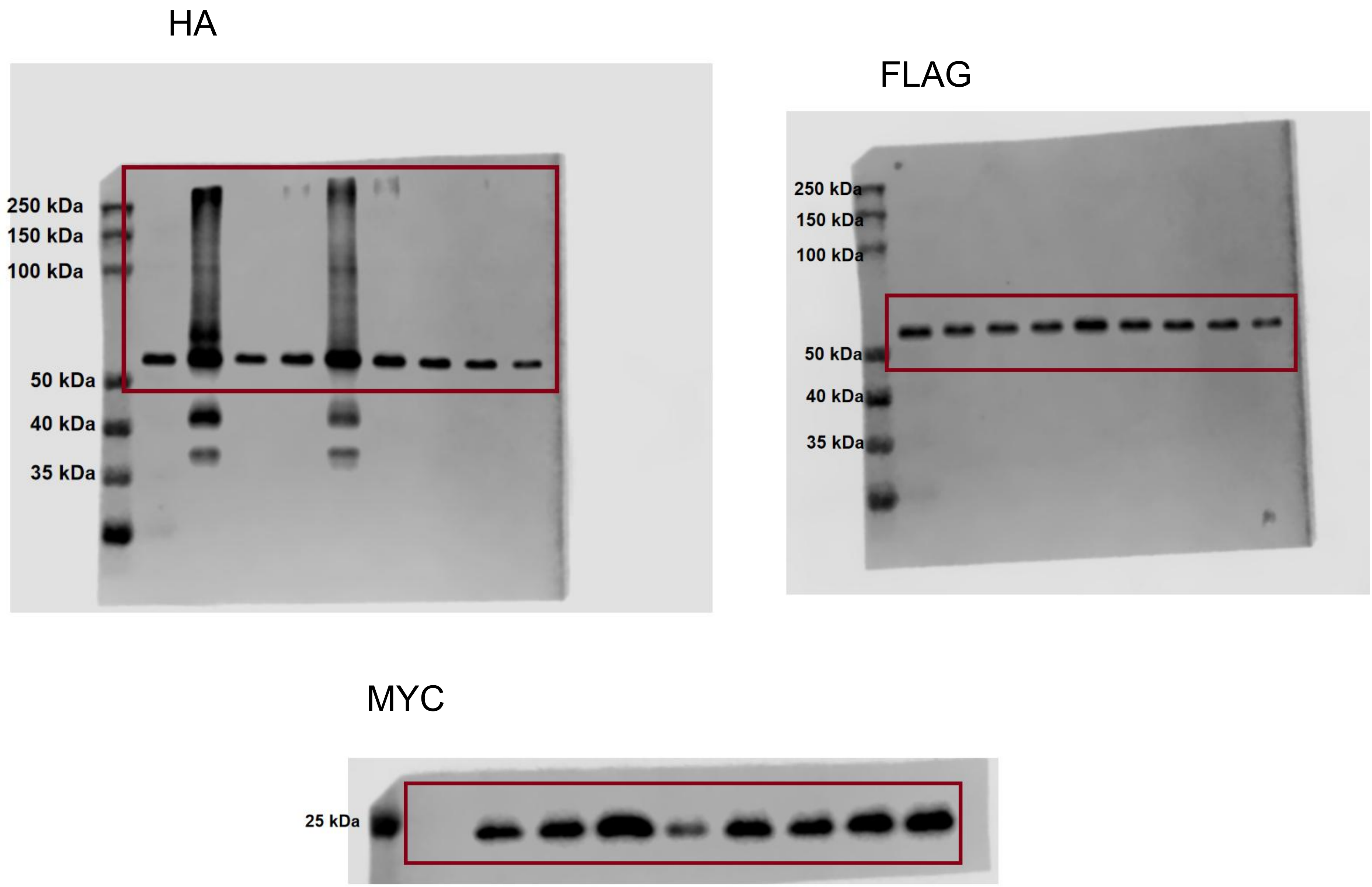
HA
250 kDa
150 kDa
100 kDa
50 kDa
40 kDa
35 kDa
FLAG
250 kDa
150 kDa
100 kDa
50 kDa
40 kDa
35 kDa
MYC
25 kDa

Fig. 13

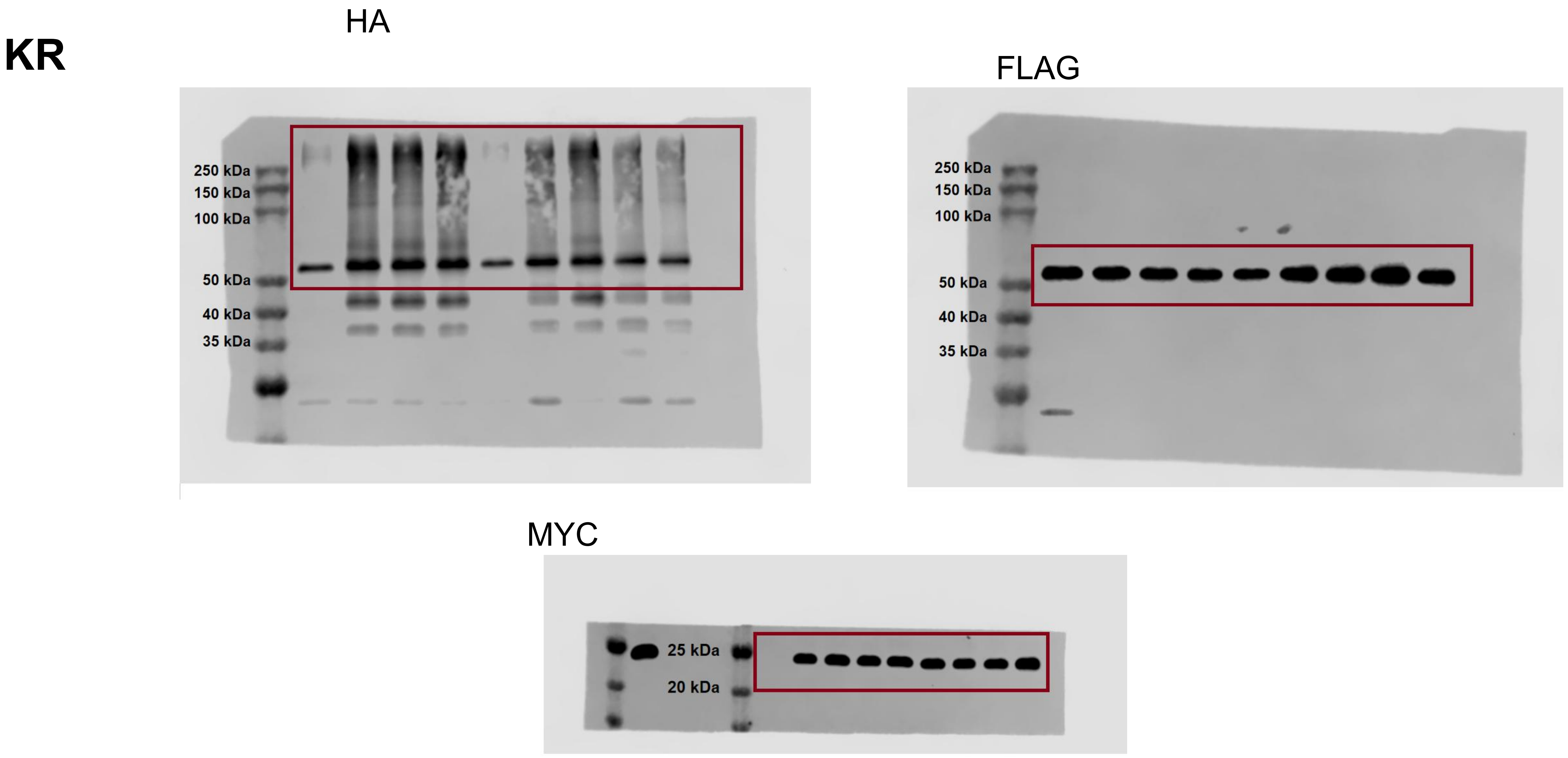

KR
HA
250 kDa
150 kDa
100 kDa
50 kDa
40 kDa
35 kDa
FLAG
250 kDa
150 kDa
100 kDa
50 kDa
40 kDa
35 kDa
MYC
25 kDa
20 kDa

Fig. S15c

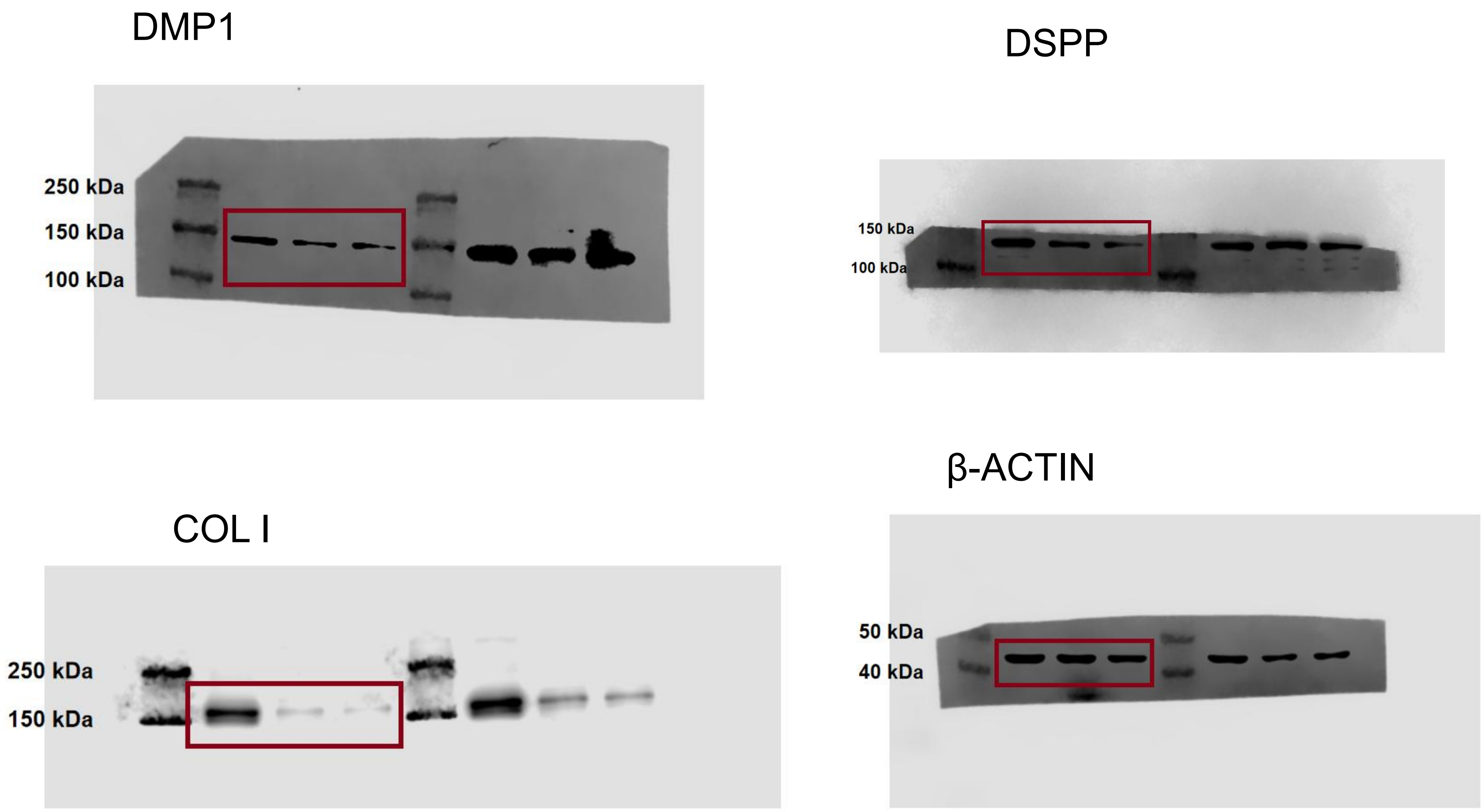
DMP1
250 kDa
150 kDa
100 kDa
DSPP
150 kDa
100 kDa
COL I
250 kDa
150 kDa
β-ACTIN
50 kDa
40 kDa
